# Diabetes-associated breast cancer is molecularly distinct and shows a DNA damage repair deficiency

**DOI:** 10.1172/jci.insight.170105

**Published:** 2023-12-08

**Authors:** Gatikrushna Panigrahi, Julián Candia, Tiffany H. Dorsey, Wei Tang, Yuuki Ohara, Jung S. Byun, Tsion Zewdu Minas, Amy Zhang, Anuoluwapo Ajao, Ashley Cellini, Harris G. Yfantis, Amy L. Flis, Dean Mann, Olga Ioffe, Xin W. Wang, Huaitian Liu, Christopher A. Loffredo, Anna Maria Napoles, Stefan Ambs

**Affiliations:** 1 Laboratory of Human Carcinogenesis, Center for Cancer Research, National Cancer Institute (NCI), NIH, Bethesda, Maryland, USA.; 2 Longitudinal Studies Section, Translational Gerontology Branch, National Institute on Aging, NIH, Baltimore, Maryland, USA.; 3 Data Science & Artificial Intelligence, R&D, AstraZeneca, Gaithersburg, Maryland, USA.; 4 Division of Intramural Research, National Institute of Minority Health and Health Disparities, NIH, Bethesda, Maryland, USA.; 5 Department of Pathology, University of Maryland Medical Center, Baltimore, Maryland, USA.; 6 Department of Pathology, University of Maryland Medical Center and Veterans Affairs Maryland Care System, Baltimore, Maryland, USA.; 7 Liver Cancer Program, Center for Cancer Research, NCI, NIH, Bethesda, Maryland, USA.; 8 Cancer Prevention and Control Program, Lombardi Comprehensive Cancer Center, Georgetown University Medical Center, Washington, DC, USA.

**Keywords:** Metabolism, Oncology, Breast cancer, Diabetes

## Abstract

Diabetes commonly affects patients with cancer. We investigated the influence of diabetes on breast cancer biology using a 3-pronged approach that included analysis of orthotopic human tumor xenografts, patient tumors, and breast cancer cells exposed to diabetes/hyperglycemia-like conditions. We aimed to identify shared phenotypes and molecular signatures by investigating the metabolome, transcriptome, and tumor mutational burden. Diabetes and hyperglycemia did not enhance cell proliferation but induced mesenchymal and stem cell–like phenotypes linked to increased mobility and odds of metastasis. They also promoted oxyradical formation and both a transcriptome and mutational signatures of DNA repair deficiency. Moreover, food- and microbiome-derived metabolites tended to accumulate in breast tumors in the presence of diabetes, potentially affecting tumor biology. Breast cancer cells cultured under hyperglycemia-like conditions acquired increased DNA damage and sensitivity to DNA repair inhibitors. Based on these observations, we conclude that diabetes-associated breast tumors may show an increased drug response to DNA damage repair inhibitors.

## Graphical abstract

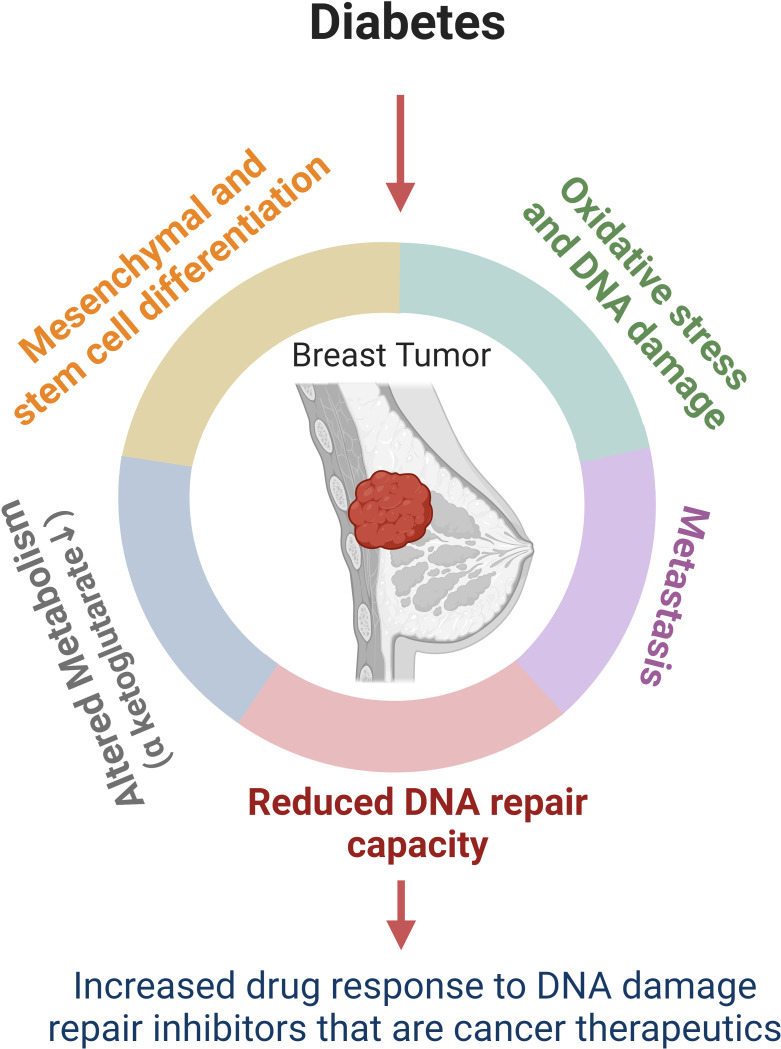

## Introduction

Comorbidities like diabetes adversely affect patients with cancer with an increasing frequency (1, 2). They negatively and disproportionately affect underserved populations and may alter tumor biology and metastasis and the choice of treatment (3). Diabetes in patients with breast cancer is linked to an increased mortality (4, 5). In African American women, diabetes is associated with decreased breast cancer survival in patients independent of the tumor estrogen receptor (ER) status (6).

Diabetes is thought to promote cancer development and progression through hyperglycemia, altered insulin signaling, and excessive inflammation (7, 8). Metabolic health, rather than obesity, might be relevant for breast cancer risk stratification (9). Although studies that investigated the diabetes-induced tumor biology in patients with breast cancer remain sparse (10), multiple investigations have described the effect of hyperglycemia and diabetes in mouse models of breast cancer (10–14). In the 4T1 mouse model of breast cancer metastasis, hyperglycemia impaired tumor vascularization but enhanced metastatic seeding due to impaired secretion of granulocyte CSF and impaired neutrophil mobilization at the metastatic site (12). Other observations show that diabetes and hyperglycemia alter the human gut microbiome and induce intestinal barrier dysfunction and enhance the risk for infections (15). We previously reported that microbiome-derived metabolites can accumulate in breast tumors (16). Therefore, we hypothesized that diabetes may influence tumor biology in patients with breast cancer by mechanisms that may include the microbiome. Tumors in patients with diabetes may acquire distinct molecular signatures that alter disease aggressiveness and therapy response.

To examine how diabetes affects breast cancer biology, we used a discovery approach consisting of 3 human xenograft models for breast cancer that were orthotopically grown in diabetes-prone NRG-Akita mice. We investigated the tumor metabolome and transcriptome and then compared the contrasts between hyperglycemic and control mice with the contrasts in human breast tumors, comparing patients with diabetes and patients without diabetes. In addition, we cultured human breast cancer cell lines under hyperglycemia for further discovery and performed mechanistic studies to validate observations. Using this approach, we identified coherent biological differences related to hyperglycemia and diabetes in both ER^–^ and ER^+^ breast cancer. Notably, our findings support the hypothesis that diabetes-associated breast tumors acquire a proinflammatory metabolome and a condition of DNA repair deficiency. Based on these observations, these tumors may show an increased response to DNA repair pathway inhibitors, which should be examined in clinical studies.

## Results

### Study design.

The effects of diabetes and hyperglycemia on breast cancer biology have not been thoroughly investigated using clinical samples. Hyperglycemia is a hallmark of type 1 and type 2 diabetes, whereas insulin secretion is reduced or absent when diabetes is established (Supplemental Figure 1, A and B; supplemental material available online with this article; https://doi.org/10.1172/jci.insight.170105DS1) (17). We applied a 3-pronged approach to obtain a comprehensive assessment of diabetes-induced effects in xenograft breast tumors, patient tumors, and human breast cancer cell lines, as outlined in Supplemental Figure 1C. Our animal model for diabetes/hyperglycemia was NRG-Akita mouse based. We used female Akita mice that progressively develop hyperglycemia with an onset at 4 weeks of age as a model of genetically induced hyperglycemia with similarities to type 1 diabetes in disease origin but exhibiting some phenotypes of type 2 diabetes (18, 19). Fresh-frozen patient tumors were obtained from women with both type 1 diabetes (*n* = 6) and type 2 diabetes (*n* = 34). Most of these women were self-identified African Americans (*n* = 33), including all patients with type 1 diabetes. African American women are a high-risk group for aggressive forms of breast cancer and are generally more affected by diabetes than other women (6, 20).

Initially, we examined the hyperglycemia-induced biology of orthotopically grown tumors from 3 ER^–^ human breast cancer cell lines, MDA-MB-231, MDA-MB-468, and Hs578T, injected into the abdominal mammary fat pad of either diabetes-prone Akita mice (NOD.Cg-Rag1^tm1Mom^ Ins2^Akita^ Il2rg^tm1Wjl^/SzJ) or its matched control (NOD.Cg-Rag1^tm1Mom^ Il2rg^tm1Wjl^/SzJ), all 8 weeks old. Tumor-bearing mice were sacrificed at 5 weeks for MDA-MB-231, 6 weeks for MDA-MB-468, or 8 weeks for Hs578T xenografts, as shown in Supplemental Figure 2A. At these time points, all Akita mice, but none of the controls, had developed hyperglycemia (*n* = 4–5 per group; Supplemental Figure 2B). Xenografts in hyperglycemic and control mice did not show significant differences in either tumor growth (Supplemental Figure 2C) or their proliferation score (Supplemental Figure 2D) across the 3 models. However, for the 1 cell line known to produce metastases from orthotopically grafted tumors, MDA-MB-231, we detected metastatic lesions in the spleen, kidney, and upper gastrointestinal tract in 2 of 4 tumor-bearing Akita mice (50%) but not in any of the 5 tumor-bearing control mice (Supplemental Figure 2E). Consistent with the xenograft growth data, hyperglycemia did not enhance proliferation in human breast cancer cells, irrespective of whether or not mannitol was added in the control experiments to adjust for osmolarity (Supplemental Figure 2F).

### Hyperglycemia-induced metabolic alterations in breast tumor xenografts.

**Figure 1:**
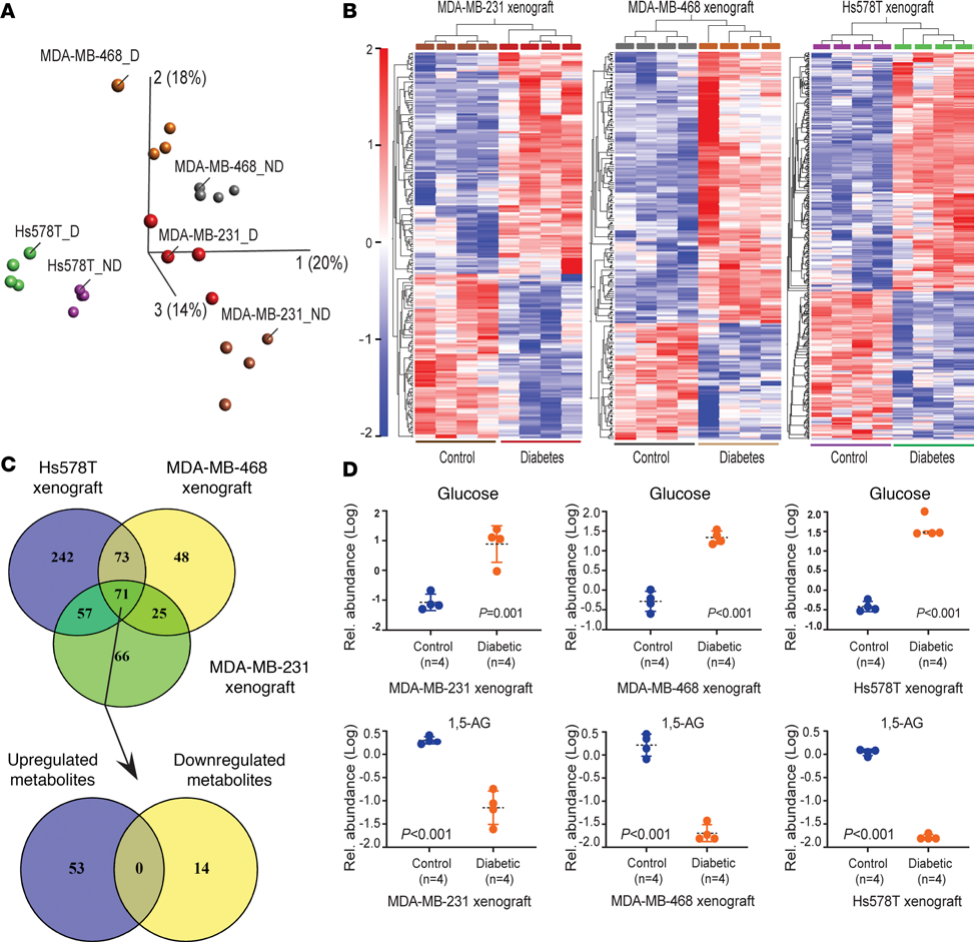
Hyperglycemia induces robust metabolite alterations in tumor xenografts. (**A**) Unsupervised PCA using the metabolite data obtained from xenografts grown in diabetic (_D) and nondiabetic mice (_ND). The plot shows data points for each of the MDA-MB-231, MDA-MB-468, and Hs578T xenografts and highlights the separation by diabetes status. (**B**) Heatmaps emphasizing the difference in intratumor metabolite abundance between diabetic and nondiabetic xenografts (FDR cutoff < 0.3 for inclusion of differential metabolites). The plots show the data from MDA-MB-231, MDA-MB-468, and Hs578T xenografts. (**C**) Venn diagram with 71 metabolites with levels altered by diabetes across MDA-MB-231, MDA-MB-468, and Hs578T xenografts (FDR < 0.05). Fifty-three of them were consistently upregulated, and 14 were downregulated in all xenografts of diabetic mice. (**D**) Intratumor levels of the diabetes markers, glucose, and 1,5 anhydroglucitol (1,5 AG), in MDA-MB-231, MDA-MB-468, and Hs578T xenografts by diabetes status. Data represent mean ± SD of log transformed relative abundance levels (*n* = 4 each group), with Student’s *t* test for significance testing.

Next, we investigated the metabolome profiles of the xenografts comparing tumors from hyperglycemic versus control mice (*n* = 4, each comparison group). Being able to detect up to 830 metabolites with the applied platform (Metabolon), we uncovered hyperglycemia-associated alterations in the tumor metabolome (Supplemental Table 1), as shown by the principal component analysis (PCA; including all metabolites) (Figure 1A) and a hierarchical cluster analysis that included all metabolites at a FDR < 0.3 (MDA-MB-231, *n* = 219; MDA-MB-468, *n* = 217; Hs578T, *n* = 443 metabolites; Figure 1B). These metabolic differences were also found in blood samples, with microbiome-derived 3-phenylpropionate and hippurate being the most upregulated serum metabolites in the presence of hyperglycemia, as shown for Akita mice bearing MDA-MB-468 xenografts (*n* = 4; Supplemental Table 1 and Supplemental Figure 3, A–D). Still, the hyperglycemia-associated metabolome contrasts for serum and tumor showed differences, with 341 metabolites being distinctively altered by hyperglycemia in serum and 91 metabolites in tumor xenografts, applying an FDR < 0.3 (Supplemental Figure 4). Across the 3 xenograft models, 71 tumor metabolites were commonly altered in the Akita mice (Figure 1C and Supplemental Table 2). Glucose was upregulated in all tumors of these mice, whereas intratumor 1,5-anhydroglucitol (1,5-AG), a known diabetes marker that is downregulated in the presence of hyperglycemia (21, 22), was greatly diminished (average 50-fold), consistent with diabetes/hyperglycemia-induced reprogramming of tumor metabolism (Figure 1D and Supplemental Figure 3, E and F). This observation was further confirmed with the concurrent accumulation of fructosyllysine (all xenografts) and N6-carboxylmethyllysine (CML) in MDA-MB-468 and Hs578T xenografts. Both metabolites belong to the family of food-derived, proinflammatory, and promutagenic advanced glycation end products that are commonly elevated in people with diabetes (23). Among the 71 tumor metabolites, 67 metabolites were either steadily increased (*n* = 53) or decreased (*n* = 14) in Akita mice (Figure 1C), with an FDR < 0.05 for each metabolite in the combined analysis across the 3 xenograft models (Supplemental Table 2). Notably, many of them represent food- or microbiome-derived metabolites that mostly accumulated in the tumors in presence of diabetes, whereas a small number of metabolites represent typical energy or tumor metabolism–related molecules (e.g., α-ketoglutarate, glucose). Food-derived metabolites include isoflavones like genistein and daidzein sulfate that may reduce the risk of breast cancer recurrence but may also interfere with the antitumor effects of breast cancer therapeutics (24, 25). At least 8 of the 71 diabetes/hyperglycemia-associated metabolites have previously been linked to the gut microbiome (26, 27) and included hippurate as the metabolite with the most significant accumulation, besides imidazole propionate, 3-phenylprorionate, or phenyl sulfate, among others (Supplemental Table 2). Hippurate, imidazole propionate, and phenyl sulfate have been reported to be increased in diabetes (26, 28, 29), consistent with our data. Last, we noticed that α-ketoglutarate, a key metabolite in the regulation and maintenance of the epigenome, was consistently downregulated in tumors of hyperglycemic mice. Interestingly, a loss of α-ketoglutarate–dependent lysine demethylase activity has recently been linked to a suppression of DNA repair by disrupting local chromatin signaling (30, 31).

### The metabolome of diabetes-associated patient tumors.

Having observed that diabetes-related hyperglycemia alters the metabolome of human breast tumor xenografts, we asked whether similar metabolic alterations can be detected in breast tumors from patients with diabetes. We analyzed the metabolome of tumors from 40 women with diabetes and 48 without diabetes and used the tumor ER status to match patients (Supplemental Table 3). Most of these patients were self-identified African American women. Patients with diabetes were older (65 versus 51.5 years of age) and tended to have a higher BMI (32.2 versus 29.2). Even so, independent of the diabetes status, most women in our patient cohort would best be categorized as overweight to obese, representing US trends for women in this age group. The metabolome contrast comparing patients with or without diabetes was consistent with the metabolome contrast in our experimental model of hyperglycemia — however less distinct — likely because patients were heterogenous with regard to their dietary intake and with regard to being treated with antidiabetic drugs. Nevertheless, when we choose an unadjusted *P* < 0.05 as the cutoff, a metabolome profile emerged that was consistent with findings in our xenograft-based discovery cohort (Supplemental Figure 3, G–I). Patients with diabetes presented with reduced intratumor 1,5-AG levels and an accumulation of microbiome-derived metabolites in their tumors, including trimethylamine N-oxide, imidazole propionate, cresol sulfate, and phenyl sulfate. Food-derived metabolites included the advanced glycation end product, CML. The intratumor accumulation of CML in patients with diabetes was robust and remained statistically significant, when compared with nondiabetic patients, after further adjustments for age, race, BMI, tumor stage, and tumor ER status. CML also accumulated in the tumor xenografts and has been described as a candidate ligand of the receptor for advanced glycation end products (RAGE) receptor that has candidate oncogenic and proinflammatory functions in cancer (32). We examined whether CML at the physiological concentration of 1 μM (33) would induce a proinflammatory RAGE signaling signature in MDA-MB-231 breast cancer cells. As shown in Supplemental Figure 5, A–C, CML induced such a signature with increased NF-κB–mediated TNF-α signaling as the top-ranked pathway. Other activated pathways included Myc and TGF-β signaling, upregulation of reactive oxygen species (ROS), the inflammatory response, and epithelial-to-mesenchymal transition (EMT).

### Diabetes-induced transcriptome is consistent with the activation of developmental pathways and a decreased DNA repair capacity.

**Figure 2:**
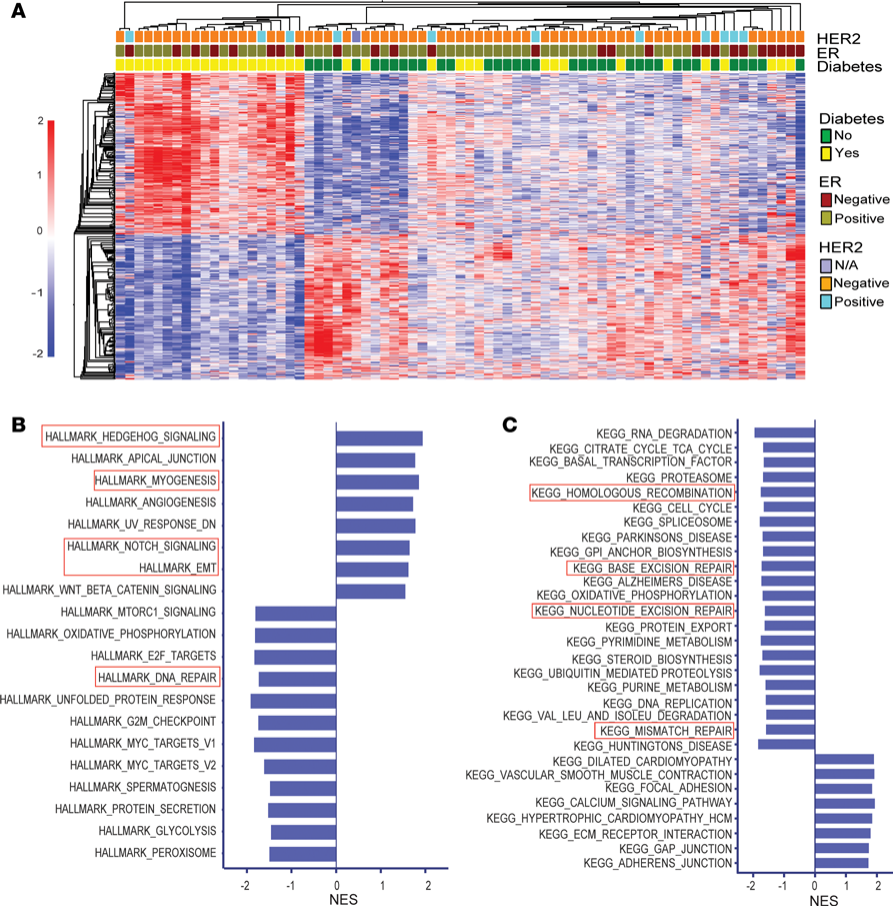
A distinct transcriptome profile in breast tumors of patients with diabetes. (**A**) Heatmap highlighting the difference in gene expression for breast tumors from diabetic (yes) and nondiabetic (no) patients (FDR < 0.05 for inclusion of differentially expressed transcripts, covariate adjusted). (**B**) Enrichment of differentially expressed genes (diabetic versus nondiabetic, covariate adjusted) in GSEA Hallmark gene sets (FDR < 0.25). The *y* axis represents the enriched gene sets (either positive or negative), and the *x* axis represents the normalized enrichment scores (NES) for each gene set. (**C**) Enrichment of differentially expressed genes (diabetic versus nondiabetic, covariate adjusted) in GSEA KEGG gene sets (FDR < 0.25). Red boxes highlight key pathways that are altered by diabetes and described in the text.

To continue our interrogation of the diabetes-associated tumor biology, we generated RNA-Seq–based gene expression profiles for the 3 tumor xenograft models, 73 patient tumors, and 6 human breast cancer cell lines cultured under hyperglycemic conditions (ER^+^: MDA-MB-175, ZR-7530, and HCC1500; ER^–^: MDA-MB-157, MDA-MB-231, and MDA-MB-468; all with exception of MDA-MB-231 being cell lines from African American donors). The 73 patient tumors were obtained from 36 patients with diabetes and 37 without diabetes and represent a subset of the tumors with metabolome data (Supplemental Table 3). Comparing the gene expression profiles between tumors from patients with diabetes versus patients without diabetes revealed large differences with 2012 differentially expressed genes at an FDR < 0.05 (Supplemental Table 4). A hierarchical cluster analysis using the 673 differential genes at an FDR < 0.05 — with additional covariate adjustments for age, race, BMI, tumor stage, and tumor ER status in generating the contrast — achieved a separation into tumors from patients with diabetes and patients without diabetes (Figure 2A), indicating that the effect of diabetes on tumor biology is well captured by the tumor transcriptome. This finding was replicated in xenografts and cultured cells. In Akita mice, experimental diabetes induced robust gene expression signatures in MDA-MB-231 and MDA-MB-468, but not Hs578T, tumor xenografts (Supplemental Figure 6, A–E). Similarly, the gene expression profiles of breast cancer cell lines cultured under hyperglycemia showed robust changes (Supplemental Figure 6, F–M).

**Figure 3:**
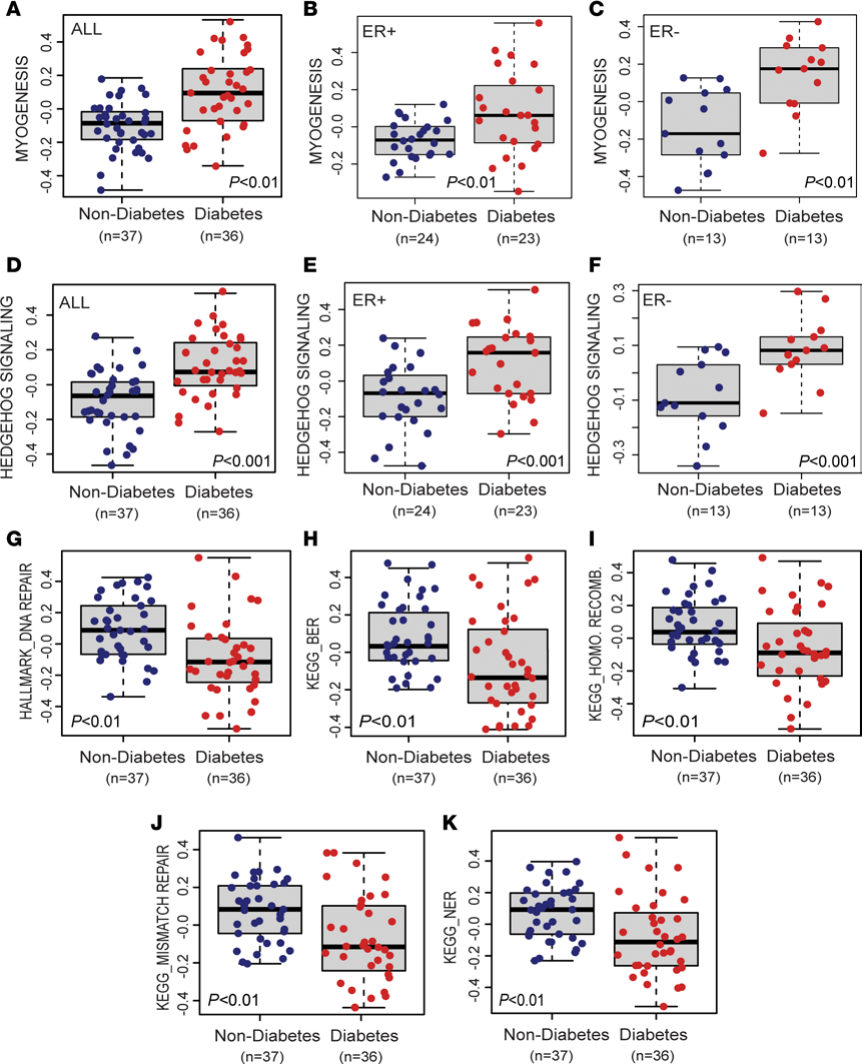
Activity scores of key pathways altered in breast tumors of patients with diabetes. (**A**–**F**) Myogenesis and hedgehog signaling score in either all, ER^+^, or ER^–^ tumors by diabetes status. (**G**–**K**) Hallmark DNA repair (**G**), KEGG Base Excision Repair pathway (BER) score (**H**), KEGG Homologous Recombination (**I**), KEGG Mismatch Repair (**J**), and KEGG Nucleotide Excision Repair (NER) pathway scores (**K**) in breast tumors by diabetes status. (**A**–**K**) Single-sample pathway scores were obtained from ssGSEA with adjustments for covariates (age, BMI, race, stage, and ER status). The significance of the diabetes status in influencing the activity scores was assessed via multivariable linear regression to control for covariates.

Next, we applied gene set enrichment analysis (GSEA) (34) using the Hallmark and KEGG pathway gene set collections to initially interrogate patient tumors (Figure 2, B and C, and Supplemental Table 5). The examination was performed with the covariate-adjusted gene list for the contrast diabetic versus nondiabetic (covariates: age, BMI, race, disease stage, tumor ER status) and revealed diabetes-associated gene signatures that included the induction of a hedgehog- and myogenesis-related gene expression program and increased notch and EMT signaling among the top-ranked pathways, pointing to a common activation of developmental and oncogenic pathways in tumors of people with diabetes (all FDR < 0.25). Myogenesis is critical to muscle development and has close links to hedgehog signaling. Hallmark myogenesis has recently been linked to a high-risk breast cancer subtype of increased mobility (35). Myogenesis and hedgehog signaling were found to associate with poor prognosis in breast cancer by these authors (35). We followed up on this finding with an analysis using single-sample GSEA–based (ssGSEA-based) pathway scores (Supplemental Methods) that capture pathway activation in individual breast tumors. With this approach, we could further demonstrate that myogenesis and hedgehog signaling are upregulated in breast tumors from patients with diabetes, largely independent of the tumor ER status (Figure 3, A–F). We also found that the myogenesis-related gene expression program was induced by diabetes and hyperglycemia in all human tumor xenografts and breast cancer cell lines, among the top-ranked pathways, whereas the hyperglycemia-induced EMT signature was most notable in the ER^+^ cell lines (Supplemental Figure 7, A–G, and Supplemental Table 6). Inflammation-related pathways, including increased TNF-α signaling, were commonly activated in both tumor xenografts and breast cancer cell lines when exposed to hyperglycemia.

GSEA Hallmark pathway enrichment scores revealed that the DNA repair capacity might be reduced in human breast tumors from patients with diabetes (Figure 2B). We followed up on this observation using GSEA KEGG pathway assignments that better define DNA repair pathways (Figure 2C) and generated pathway activity scores for individual tumors. This line of inquiry provided further indication of a broadly reduced DNA repair capacity in patient tumors (Figure 3, G–K) and human xenografts (Supplemental Figure 8A and Supplemental Table 7). Downregulation of the repair pathways in patient tumors occurred independently of the tumor ER status (Supplemental Figure 8, B and C). Most prominent was the deficiency in KEGG pathway–annotated base excision repair, mismatch repair, and homologous recombination. We observed a similar downregulation of DNA repair pathways in breast cancer cell lines (Supplemental Table 7). A homologous recombination deficiency typically associates with “BRCAness” of breast tumors due to mutational inactivation of the *BRCA1* and *BRCA2* tumor suppressor genes, making these tumors susceptible to poly-ADP ribose polymerase (PARP) inhibitors. However, diabetes may induce “BRCAness” by an alternative mechanism involving loss of gene expression, since *BRCA1* and *BRCA2* transcripts were commonly downregulated in the clinical samples and xenografts in the presence of diabetes (Supplemental Figure 9, A and B).

Finally, we performed an integration of transcriptome and metabolome data to identify candidate pathways that may have a key function in defining the effects of diabetes on tumor metabolism. To do so, we combined the transcriptome and metabolome data from across the 24 breast tumor xenografts using, first, a correlation analysis followed by GSEA, with genes ranked by their correlation coefficient. We restricted this analysis to correlations of gene expression with the 67 metabolites that were consistently increased (*n* = 53) or decreased (*n* = 14) in tumors of diabetic Akita mice (Supplemental Table 2). This exploratory approach showed that myogenesis and mitochondrial oxidative phosphorylation as the top-ranked pathways may mediate the effect of diabetes on tumor metabolism in these xenografts (Supplemental Table 8).

### Diabetes and hyperglycemia induce an invasive phenotype.

**Figure 4:**
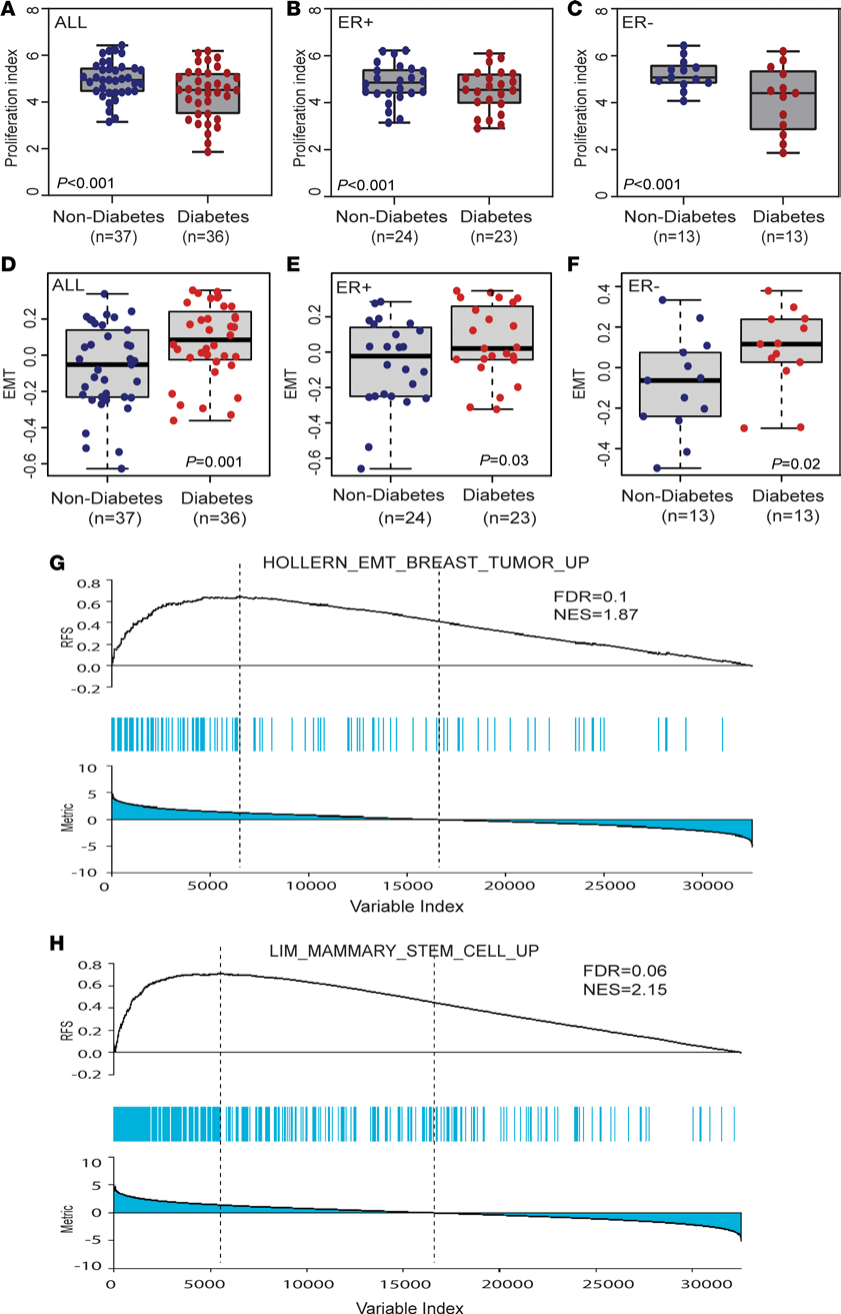
Diabetes and hyperglycemia promote mesenchymal and stem cell differentiation. (**A**–**C**) Tumor proliferation index in ER^+^ and ER^–^ breast tumors by diabetes status. Significance testing with Wilcoxon’s rank-sum test. (**D**–**F**) Hallmark-annotated EMT pathway scores (ssGSEA based and covariate adjusted) in ER^+^ and ER^–^ tumors by diabetes status; Wilcoxon’s test was used. (**G**) Enrichment of differentially expressed genes (diabetic versus nondiabetic; covariate adjusted) in GSEA gene set HOLLERN_EMT_BREAST_TUMOR_UP. Signature is up with diabetes. (**H**) Enrichment of differentially expressed genes (diabetic versus nondiabetic; covariate-adjusted) in GSEA gene set LIM_MAMMARY_STEM_CELL_UP. Signature is up with diabetes.

**Figure 5:**
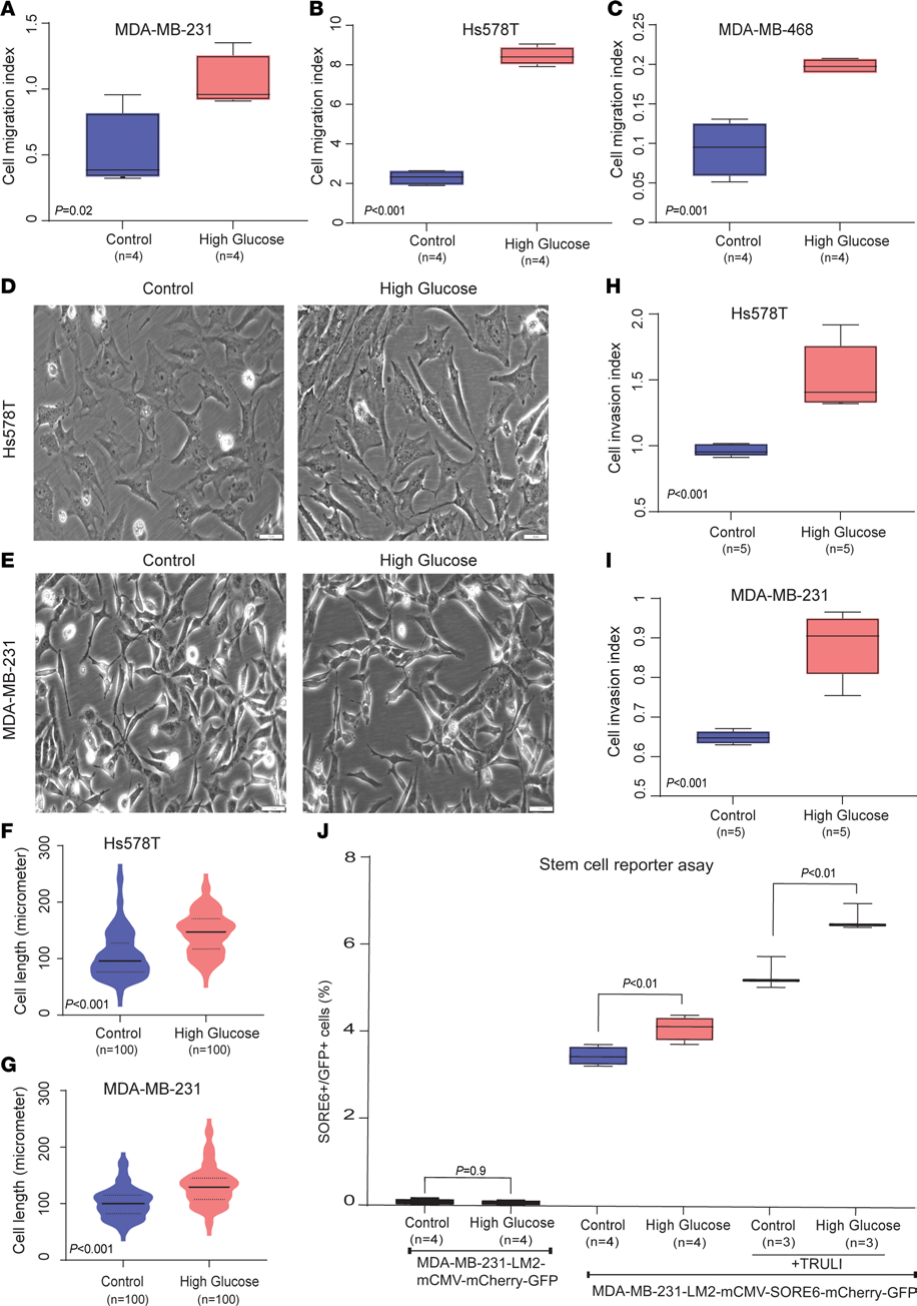
Hyperglycemia induces breast cancer cell migration, invasion, and stemness. (**A**–**C**) Migration of breast cancer cells (MDA-MB-231, Hs578T, MDA-MB-468) under hyperglycemia. Shown are data for the 24-hour time point. Data represent mean ± SD of 4 replicates; Student’s *t* test was used. (**D** and **E**) Hs578T and MDA-MB-231 cells cultured under hyperglycemia develop an elongated morphology. Total original magnification, ×200. The scale bar is 100 µm. (**F** and **G**) Quantitative analysis of the elongated cell morphology in Hs578T and MDA-MB-231 cells cultured under hyperglycemia using the ImageJ software (NIH). Data represent average length of 100 cells from 5 different representative areas in each group; Wilcoxon’s test was used. (**H**) Matrigel invasion by Hs578T breast cancer cells under hyperglycemia. Shown are data for the 24-hour time point. Data represent mean ± SD of 5 replicates; Student’s *t* test was used. (**I**) Matrigel invasion by MDA-MB-231 breast cancer cells under hyperglycemia. Shown are data for the 24-hour time point. Data represent mean ± SD of 5 replicates; Student’s *t* test was used. (**J**) MDA-MB-231-LM2 cells harboring a stemness reporter were cultured with or without hyperglycemia. Number of SORE6^+^ cells among cultured MDA-MB-231-LM2-SORE6-mcherry breast cancer cells exposed to either 5 mM glucose (control) or hyperglycemia (25 mM glucose) for 48 hours. Hyperglycemia increases the number of SORE6^+^ cells, which is indicative of increased stemness. Addition of the positive control compound, TRULI, a Lats1/2 kinase inhibitor, increases the stemness signal. We did not observe SORE6^+^ cells among the control vector cells (MDA-MB-231-LM2-mCMV-mcherry) when cultured with or without 25 mM glucose. Data represent mean ± SD; Student’s *t* test was used for statistical analysis.

Diabetes did not significantly influence tumor xenograft growth (Supplemental Figure 2, C and D) but may promote metastasis, as shown for the implanted MDA-MB-231 cells (Supplemental Figure 2E and Supplemental Figure 10A). Thus, we assessed the effect of diabetes on both cell proliferation and a prometastatic phenotype in patient tumors using the transcriptome data and examined how hyperglycemia may affect human breast cancer cells in culture. Tumors from patients with diabetes exhibited decreased proliferation in both ER^–^ and ER^+^ tumors (Figure 4, A–C), as judged by their proliferation score derived from a validated gene expression proliferation signature (16, 36). In contrast, the Hallmark EMT signature was upregulated in breast tumors of patients with diabetes (Figure 4, D–F). Transcript levels of several EMT driver genes — e.g., *ZEB1*, *VIM*, *TWIST1* — were elevated in breast tumors of patients with diabetes (Supplemental Figure 11), and the diabetes-associated transcriptome showed significant overlap with the Hollern_EMT_Breast_Tumor_Up as well as a LIM-Mammary_Stem_Cell_Up signatures in the GSEA MSigDB collection (Figure 4, G and H; FDR = 0.1 and 0.06, respectively). To corroborate that hyperglycemia induces a mesenchymal phenotype with increased mobility, we measured migration and invasion under hyperglycemic conditions of human breast cancer cells and also determined the acquisition of a mesenchymal phenotype by quantifying cell length in culture to capture a spindle-like morphology. In agreement with the pathway analysis, hyperglycemia increased cell migration (Figure 5, A–C), but not proliferation (Supplemental Figure 2F), and induced a more spindle cell–like appearance as quantified by an increased average cell length (Figure 5, D–G). Additionally, we performed the Matrigel invasion assay, where we observed that hyperglycemia increased breast cancer cell invasion (Figure 5, H and I). To further validate that hyperglycemia may increase stemness in breast cancer cells, we cultured MDA-MB-231-LM2 cells carrying a reporter construct in which 6 concatenated repeats of a composite SOX2/OCT4 response element from the proximal human NANOG promoter are coupled to a minimal cytomegalovirus (CMV) promoter, to drive expression of a fluorescent reporter gene for stem cell signaling (37). Using this cell line, we show that our standard hyperglycemic culture conditions induce the fluorescent reporter, thereby showing increased stemness (Figure 5J). Finally, transcript levels of multiple cancer stem cell markers tended to be upregulated in breast tumors of patients with diabetes (Supplemental Figure 12).

### Hyperglycemia induces mitochondrial oxyradical formation and DNA damage and increases sensitivity to DNA repair inhibitor drugs.

**Figure 6:**
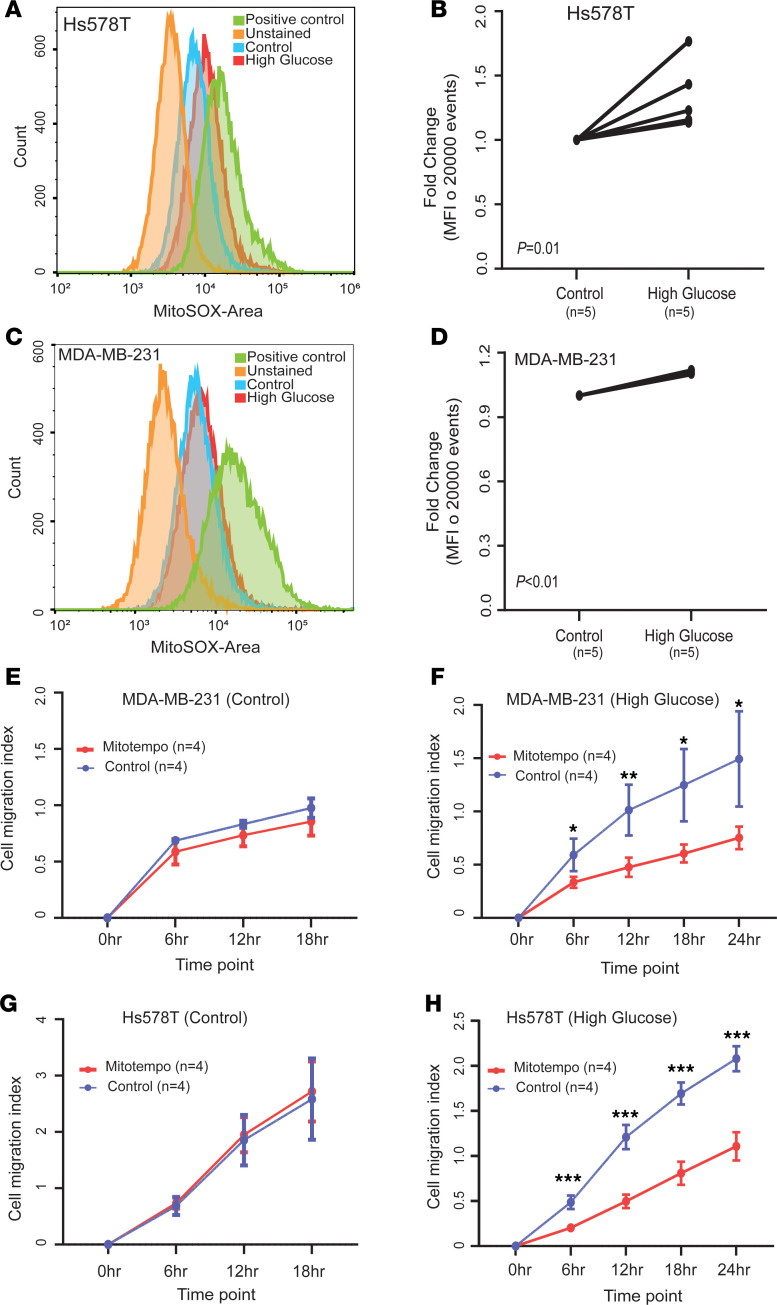
Hyperglycemia induces oxidative stress in breast cancer cells. (**A**–**D**) FACS analysis shows increased mitochondrial superoxide production (with MitoSOX) in breast cancer cells cultured under hyperglycemia. Shown are representative flow cytometry experiments for the Hs578T (**A**) and MDA-MB-231 (**C**) cell lines. There is a shift toward increased MitoSOX under hyperglycemia. Quantification of FACS analysis data for Hs578T and MDA-MB-231 cells are shown in **B** and **D**, respectively. MitoSOX fluorescence compared control versus hyperglycemia with 5 repeats; Student’s *t* test was used. For normalization and display, we set control values as 1. (**E**–**H**) Superoxide radical scavenger, Mitotempo (200 μM), inhibits hyperglycemia-induced migration of Hs578T and MDA-MB-231 cells. Each time point shows mean ± SD of 4 replicates. ANOVA with post hoc test for statistical analysis. *P < 0.05, **P < 0.01, ***P < 0.001.

**Figure 7:**
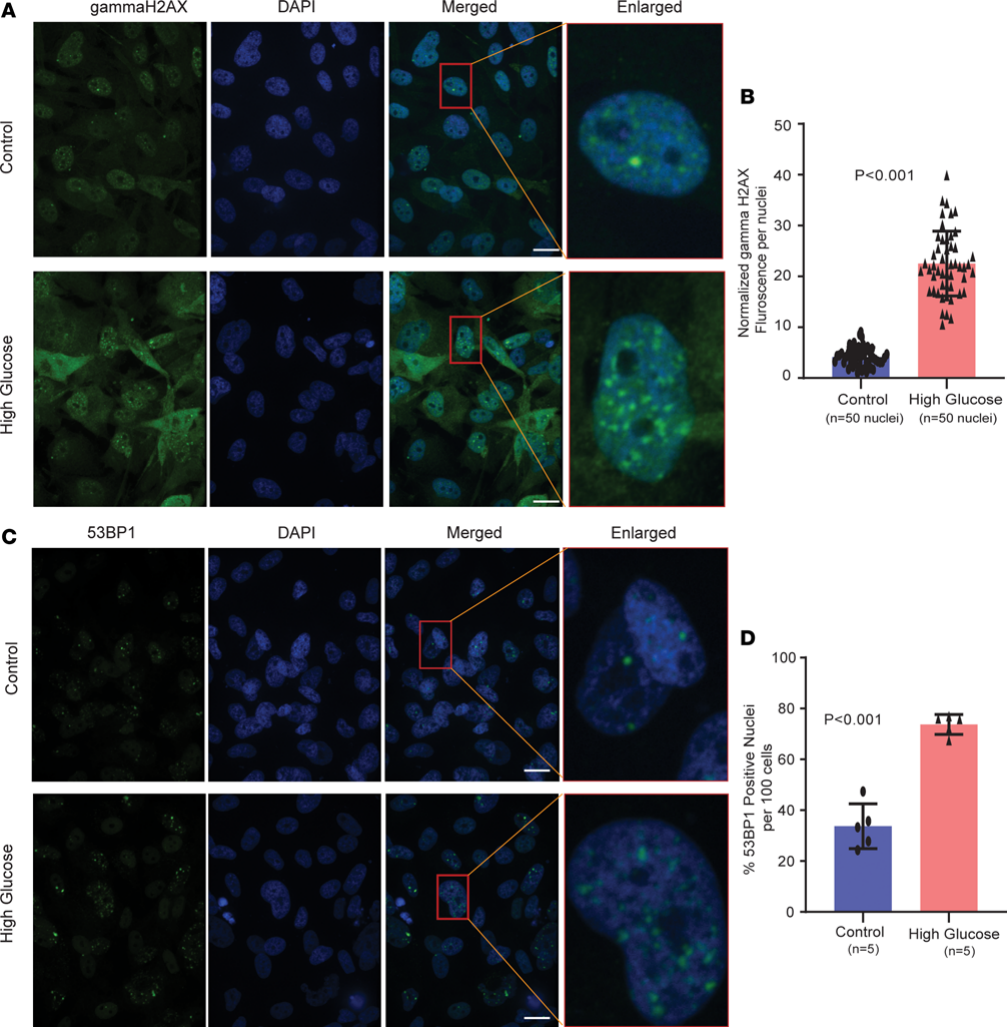
Hyperglycemia induces DNA damage in breast cancer cells. (**A**) Representative immunofluorescence images of γH2AX staining in Hs578T cells under hyperglycemia. Scale bar: 20 μm for γH2AX, DAPI, and merged images. Original magnification for the enlarged image is 25×. (**B**) Quantification of γH2AX in Hs578T cells comparing control versus hyperglycemia using ImageJ software. Data show mean ± SD of normalized fluorescence from 50 nuclei taken from 5 different areas for each group; Student’s *t* test was used for significance testing. (**C**) Representative immunofluorescence images of 53BP1 staining in Hs578T cells under hyperglycemia. Scale bar: 20 μm. Original magnification for the enlarged image is 25×. (**D**) Quantification of 53BP1 in Hs578T cells comparing control versus hyperglycemia using ImageJ software. Data represent mean ± SD of the average percentage of localized 53BP1 expression in positive nuclei in each group, using *n* = 5 images from each group and Student’s *t* test.

**Figure 8:**
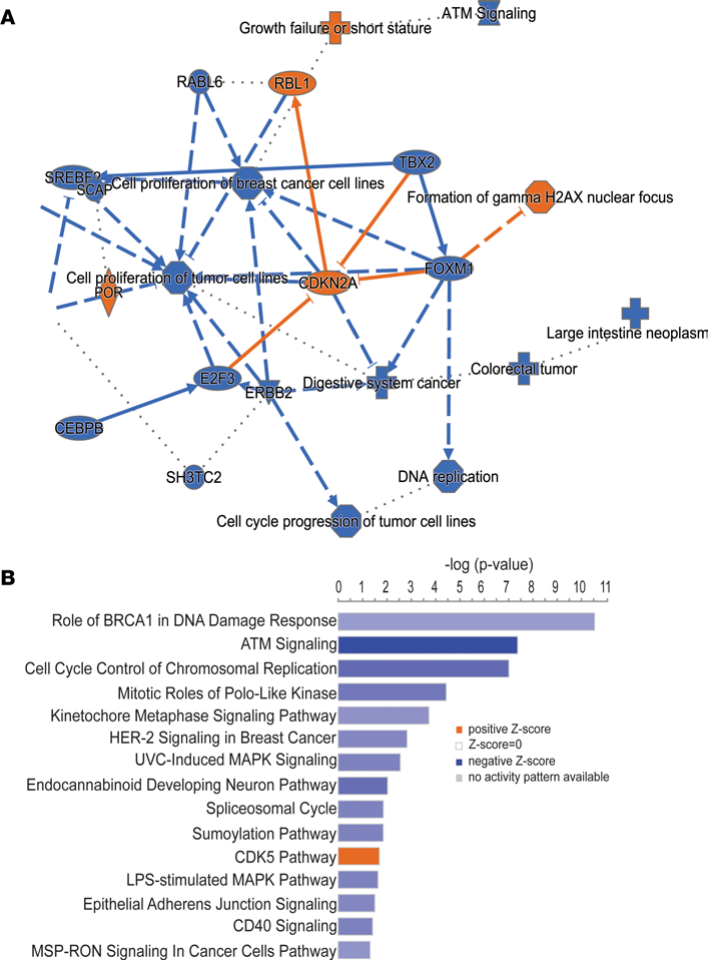
Diabetes affects DNA repair capacity, shown by IPA. IPA with 461 genes whose expression is commonly altered by diabetes/hyperglycemia in both patient tumors and xenografts. (**A**) Summary graph of the IPA indicates activation of DNA damage signaling like “Formation of gamma H2AX nuclear focus” in the presence of diabetes. Blue indicates “inhibition” and orange indicates “activation” of a process. (**B**) Pathway enrichment analyses in IPA. Blue indicates “inhibition” and orange indicates “activation” of a pathway/process by diabetes. “Role of BRCA1 in DNA damage response” is the top pathway indicated to be inhibited by diabetes.

Oxidative stress due to hyperglycemia has been linked to lung metastasis in a syngeneic mouse metastasis model (38). Hence, we asked if hyperglycemia may cause oxidative stress and whether it increases DNA damage in human breast cancer cells. We confirmed the elevated production of mitochondrial ROS by FACS (Figure 6, A–D), albeit with an estimated moderate increase, and found the ROS scavenger Mitotempo to inhibit hyperglycemia-induced cell migration, indicating that upregulation of ROS production is an inducer of migration under hyperglycemia in these cells (Figure 6, E–H). The observation is in agreement with the literature showing that moderate rather than sizable increases of ROS enhance metastasis (39). To examine whether hyperglycemia and ROS may increase DNA damage, we examined the number of cells positive for the 2 DNA damage markers, γH2AX (40) and 53BP1 (41), using immunofluorescence microscopy. Both markers were significantly elevated in Hs578T and MDA-MB-231 cells when cultured under hyperglycemia (Figure 7, A–D, and Supplemental Figure 9, C and D). In addition to nuclear staining, we also observed diffuse cytoplasmic staining of γH2AX in the presence of hyperglycemia. A previous report suggested that a DNA repair deficiency can lead to accumulation of fragments of unrepaired genomic DNA in the cytoplasm, with increased cytoplasmic γH2AX (42). This notion of increased DNA damage and a reduced DNA damage repair capacity was further supported by an Ingenuity Pathway Analysis (IPA; QIAGEN) that included 461 differentially expressed genes across patient and xenograft tumors (diabetic versus nondiabetic; Supplemental Table 9), showing that “the role of BRCA1 in DNA damage response” is the top downregulated IPA-defined process in presence of diabetes (Figure 8, A and B). The downregulation of these DNA repair pathways was also suggested when we performed another IPA with the 311 genes whose expression was similarly altered by hyperglycemia (95 upregulated and 216 downregulated, FDR < 0.3) across the MDA-MB-231 and MDA-MB-468 xenografts and their corresponding cell lines (Supplemental Figure 13, A and B, and Supplemental Table 9).

**Figure 9:**
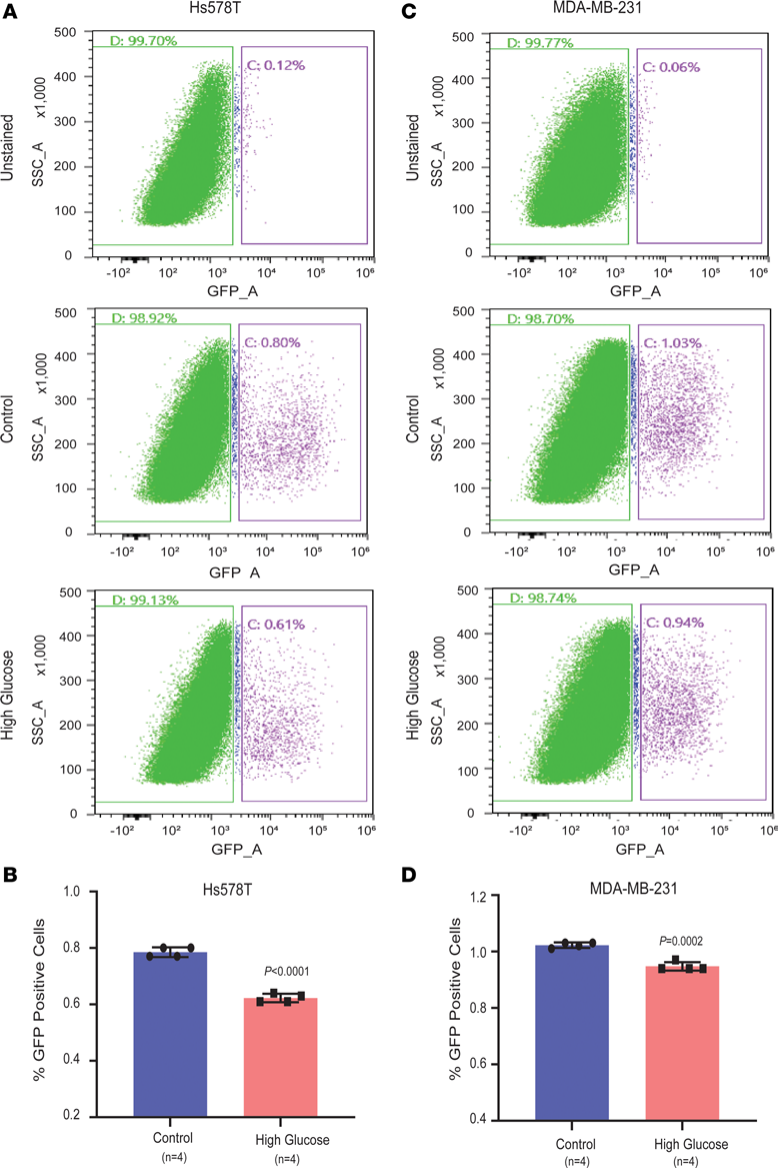
Hyperglycemia impairs DNA repair capacity in breast cancer cells. (**A**–**D**) Decreased NHEJ DNA repair capacity under hyperglycemia. Breast cancer cells (Hs578T and MDA-MB-231) cultured under high glucose showed a decrease in the nonhomologous end joining (NHEJ) DNA repair capacity, as measured by a reporter assay. A decrease in GFP^+^ cells in the high-glucose groups corresponds to a decrease in DNA repair capacity. **A** and **C** represent the FACS analysis of Hs578T and MDA-MB-231 cells, respectively. The graphs show the quantification of FACS analysis-based data for Hs578T and MDA-MB-231 cells in **B** and **D**, respectively. Data represent mean ± SD of the percent of GFP^+^ cells comparing hyperglycemia (25 mM glucose) versus control (5 mM glucose, *n* = 4 each), with significance testing by Student’s *t* test.

Furthermore, using a reporter assay to measure nonhomologous end joining (NHEJ) DNA repair capacity, we could demonstrate a decrease in NHEJ in MDA-MB-231 and Hs578T breast cancer cells when cultured under hyperglycemia (Figure 9, A–D). A downregulation of this pathway activity is consistent with partial BRCA1/2 inhibition in the presence of diabetes and hyperglycemia (Supplemental Figure 9, A and B).

We followed up on our observations with an analysis of nuclear γH2AX positivity in 105 breast tumors from 29 patients with diabetes and 76 patients who did not have a diagnosis of diabetes. γH2AX positivity in the tumor epithelium was present in 58.6% of the patients with diabetes (17 of 29) and 36.8% of the patients without diabetes (28 of 76) (*P* = 0.05, 2-tailed Fisher’s exact test). We obtained a moderate to high γH2AX immunostaining score for 44.8% of the tumors from patients with diabetes (13 of 29), whereas 30.2% of the tumors from patients without diabetes (23 of 76) scored in this range (*P* = 0.17).

**Figure 10:**
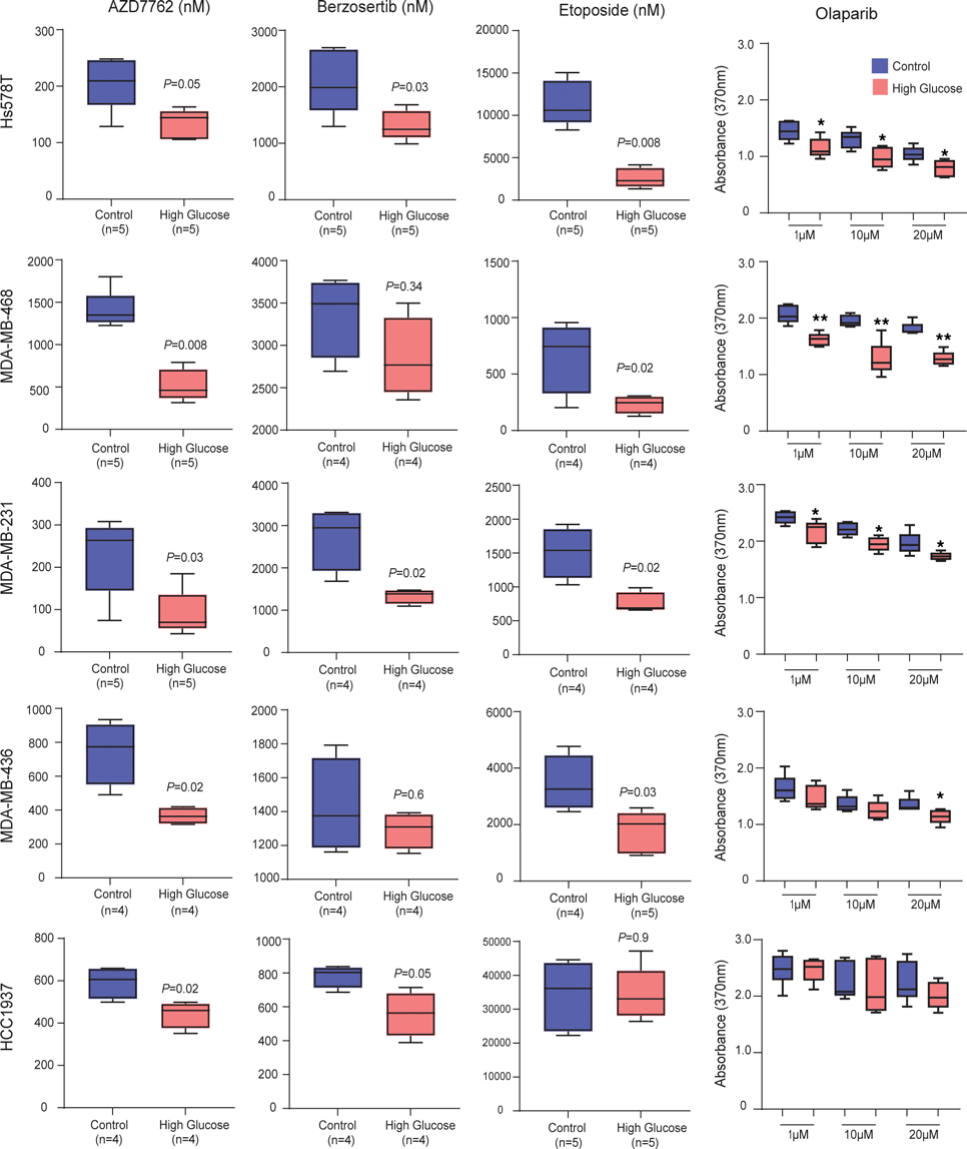
Hyperglycemia increases sensitivity to drugs targeting the DNA damage repair pathway. Increased sensitivity of 5 human breast cancer cell lines to DNA damage repair inhibitors under hyperglycemia. Shown are the IC_50_ values as nM concentrations for AZD7762, berzosertib, and etoposide comparing cells cultured under control conditions (5 mM glucose) versus high glucose (25 mM glucose [hyperglycemia]). Panel at the right shows the sensitivity to 1, 10, and 20 μM concentrations of olaparib comparing control versus high glucose with normalized BrdU incorporation (absorbance at 370 nm) as a readout. Cell viability was measured with the BrdU incorporation assay. Data are shown as mean ± SD, with Wilcoxon’s rank-sum test used for significance testing.

Because pathway analysis pointed to a broadly reduced DNA repair capacity in breast tumors of patients with diabetes, we treated multiple breast cancer cell lines (Hs578T, MDA-MB-468, MDA-MB-231, MDA-MB-436, and HCC1937) with drugs targeting DNA repair mechanisms — namely AZD7762, berzosertib, etoposide, and olaparib — and examined drug sensitivity in a BrdU incorporation–based cell proliferation assay (Figure 10). For AZD7762, berzosertib, and etoposide, we determined the IC_50_ under the control condition (5 mM glucose) and hyperglycemia (25 mM glucose), whereas for olaparib, we determined BrdU incorporation for the 1, 10, and 20 μM concentration range, since the IC_50_ tended to exceed 50 μM, in agreement with the literature (43). MDA-MB-436 and HCC1937 are cell lines that harbor mutant *BRCA1* (44). The 5 cell lines showed a generally increased sensitivity to these drugs under hyperglycemia, as defined by their decreased IC_50_ drug response values (Figure 10). For olaparib, the 2 *BRCA1* mutant cell lines had the weakest differential response under the 2 culture conditions. Similarly, when using a CellTiter-Blue–based viability assay and a defined drug concentration with 4 cell lines, we observed a decrease in viability by about 20%–30% under hyperglycemia when compared with control cells at the 48 hours exposure time point (Supplemental Figure 14).

### Diabetes associates with a distinct tumor mutational signature in patients with breast cancer.

**Figure 11:**
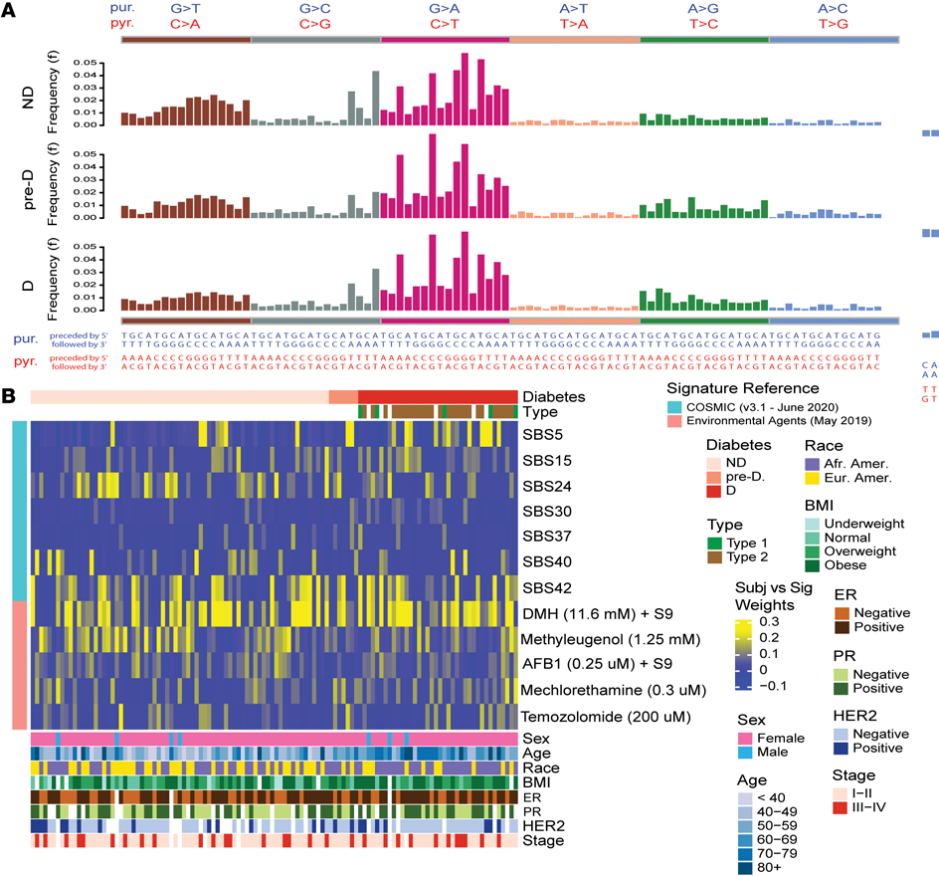
Mutational signatures in patients with breast cancer with diabetes. (**A**) Mutational trinucleotide frequency distribution in breast tumors from patients without diabetes (ND, top), with diabetes developing after the tumor resection (pre-D, center), and with diabetes at the time of tumor resection (D, bottom). Due to strand complementarity, 2 equivalent sets of annotations are possible, either based on the substitution of purines (blue) or pyrimidines (red). There are no obvious differences by diabetes status. (**B**) Heatmap showing signature age-adjusted weights by diabetes status (top bar) obtained from nonnegative least squares mapping of individual samples (columns) versus reference signatures (rows) from the COSMIC catalogs and the Compendium of Mutational Signatures of Environmental Agents. Yellow indicates upregulation of a signature in a sample. D, *n* = 38; pre-D, *n* = 7; ND, *n* = 71.

**Figure 12:**
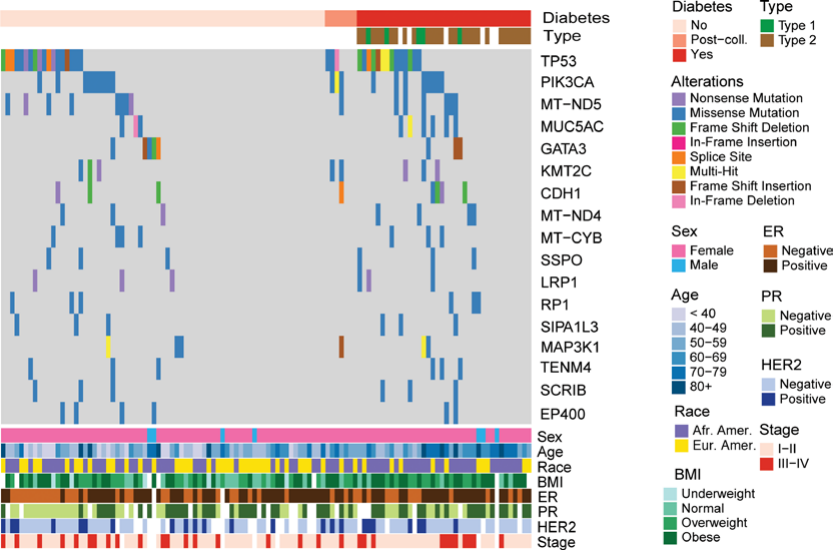
Mutational landscape of patients with breast cancer with diabetes. Oncoplot showing 17 mutated genes (rows) across 116 subjects (columns) split by diabetes status. Within each group, individuals were ordered in waterfall fashion. Included genes are those mutated in > 5% of the samples. Diabetic, *n* = 38; diabetic after surgery, *n* = 7; nondiabetic, *n* = 71. Post-coll, patients developed diabetes after tumors were collected.

Because hyperglycemia increased DNA damage in cultured cells, we asked whether breast tumors from patients with diabetes may acquire an increased mutational burden or a distinct mutational signature. Using whole-exome sequencing (WES), we analyzed 38 breast tumors from patients with diabetes, 71 tumors from patients without diabetes, and 7 tumors from patients who developed diabetes on follow-up. We did not find obvious differences in the overall frequency of somatic mutations comparing tumors by patients’ diabetes status (Figure 11A and Supplemental Table 10). However, we found that tumors from patients with diabetes contain signatures of a reduced DNA damage repair capacity (SBS5 and SBS30) related to base excision repair (Supplemental Table 10). In an analysis that applied subject/signature age-adjusted weights to control for age differences between patients with and without diabetes (45), and comparing individual samples versus reference signatures from the COSMIC catalog (46, 47) and the Compendium of Mutational Signatures of Environmental Agents (48), we could identify several signatures with a disparate prevalence among the nondiabetes and diabetes groups (2-sided Wilcoxon’s test, *P* < 0.05 in the unadjusted analysis) (Figure 11B and Supplemental Table 10). Of those 2 COSMIC signatures, SBS5 (*P* = 0.001; FDR = 0.057) and SBS30 (*P* = 0.007; FDR = 0.187) stood out by being most robustly upregulated in tumors of patients with diabetes. The frequencies of these 2 signatures were similarly elevated in ER^+^ (SBS5, *P* = 0.026; SBS30, *P* = 0.031) and ER^–^ tumors (SBS5, *P* = 0.043; SBS30, *P* = 0.024). Notably, both signatures are associated with impaired DNA repair. According to the COSMIC catalog, SBS5 was found to be increased in bladder tumors with mutations in the DNA excision repair gene *ERCC2*, whereas signature SBS30 was found to be related to a deficiency in base excision repair due to inactivating mutations in *NTHL1*. Thus, enrichment of these signatures in association with diabetes is consistent with the impairment of DNA damage repair pathways that was predicted from the transcriptome analysis. In a last analysis, we compared the fraction of mutated samples for genes with a mutation in more than 5% of the samples (Figure 12 and Supplemental Table 10). We found that several genes, including *TP53*, *PIK3CA*, *MUC5AC*, and *CDH1*, had higher mutation frequencies in tumors of patients with diabetes than patients without diabetes, with mutation frequencies for *TP53*, *PIK3CA*, *MUC5AC*, and *CDH1* being 37%, 21%, 16%, and 11%, respectively, in patients with diabetes but 25%, 13%, 4%, and 4%, respectively, in patients without diabetes. However, these differences did not reach statistical significance (Supplemental Table 10). We did not find that mutations in *BRCA1/2* or other repair genes were increased in breast tumors of patients with diabetes, suggesting that loss of gene expression rather than mutational inactivation is the underlying mechanism of the DNA repair deficiency in these tumors.

## Discussion

Diabetes commonly affects patients with breast cancer, and 1 of 6–8 women with breast cancer has diabetes as a comorbidity (4, 49). It affects African American women with breast cancer more so than European American women (6, 50). In people with diabetes, breast cancer tends to have more aggressive features and to associate with decreased patient survival (4, 6, 51). Despite the evidence that diabetes may affect disease outcome and response to therapy, we are still lacking an understanding of the diabetes-induced molecular changes in human breast tumors.

Here, we used a broad approach that included analysis of patient tumors, orthotopic human tumor xenografts, and human breast cancer cells exposed to diabetes and hyperglycemia to gain an understanding of how diabetes may alter the tumor biology in patients with breast cancer. We found that food- and microbiome-derived metabolites accumulate in breast tumors in the presence of diabetes and hyperglycemia. In contrast, α-ketoglutarate is consistently downregulated in tumors of hyperglycemic mice, and this may lead to a partial inhibition of ketoglutarate-dependent enzyme activities. Importantly, the loss of α-ketoglutarate–dependent lysine demethylase activity, namely of KDM4B, has recently been linked to a suppression of DNA repair by disrupting local chromatin signaling (30, 31).

Diabetes also induced EMT- and stem cell–like phenotypes generally linked to dedifferentiation, increased mobility, and odds of metastasis. Furthermore, and perhaps most significant, diabetes associated with gene expression and mutational signatures of a DNA damage repair deficiency. Most of these phenotypes occurred in both ER^–^ and ER^+^ breast tumors. The repair deficiency may partly relate to a diabetes-associated downregulation of BRCA1/2 function in breast tumors, as our data suggest, and to metabolic alterations that increase oxidative stress and reduce the availability of α-ketoglutarate. Still, other repair pathways that are independent of BRCA1/2 function were similarly inhibited. Correspondingly, breast cancer cells cultured under hyperglycemia acquired increased DNA damage and sensitivity to DNA damage response inhibitors. Hence, diabetes-associated breast tumors may show an augmented drug response to DNA damage repair pathway inhibitors that are cancer therapeutics.

The effect of diabetes and hyperglycemia on tumor growth and metastasis has been studied in syngeneic mouse tumor models, including the 4T1 and E0771 breast cancer models (12–14, 38). Some of these studies observed an increased tumor growth induced by diabetes (13, 14), whereas others did not (12, 38). We did not find any evidence of a growth-accelerating effect by diabetes/hyperglycemia in human breast cancer cells. In patient tumors, diabetes associated with a reduced proliferation score in both the ER^–^ and ER^+^ disease. However, consistent with the mouse tumor model, we obtained evidence of a diabetes-induced mesenchymal transition, increased migration, and increased odds of metastasis in in vitro and in vivo models of human breast cancer. Also consistent with the literature (38, 52), hyperglycemia increased mitochondrial ROS production in human breast cancer cells, with ROS being a key factor in the increased mobility of these cells. Having made these observations, one would argue that diabetes increases the odds of metastasis in humans rather than breast cancer growth. Nonetheless, there is evidence from epidemiology that diabetes not only increases mortality of patients with breast cancer (4, 6, 51) but also raises the disease risk (53).

Myogenesis is the formation of skeletal muscular tissue during embryonic development and is important in muscle tissue regeneration. The latter is commonly inhibited in patients with cancer and leads to the condition of cachexia with extreme loss of skeletal muscle tissue (54, 55). The upregulation and oncogenic role of myogenic transcription factors (*MYF5*, *MYOD*) has been described for rhabdomyosarcoma (56), a pediatric malignancy of muscle. To our understanding, myogenesis has not been recognized as an oncogenic signaling pathway that functions in epithelial cancers. However, upregulated myogenesis has recently been linked to a high-risk breast cancer subtype of increased mobility (35). In our study, myogenesis was consistently identified as the top-ranked biological process that is activated in both ER^–^ and ER^+^ breast tumors of patients with diabetes compared with patients without diabetes as well as in breast tumor xenografts and breast cancer cell line under hyperglycemia. Since Hallmark myogenesis encompasses hedgehog and notch signaling, the upregulation of this biological process by diabetes may broadly reflect the activation of developmental pathways that become oncogenic in the context of cancer and promote increased cell mobility.

We investigated the diabetes-associated metabolome using untargeted metabolomics and measured 830 metabolites, including the diabetes marker 1,5-AG in the tumor tissues. The analysis of our human xenograft data shows that diabetes and hyperglycemia influence the tumor metabolome, leading to the accumulation of food- and microbiome-derived metabolites in tumors as a key feature of diabetes-induced alteration under controlled conditions. We did not find the same robust changes in patients with breast cancer, likely because of the heterogeneity in their diet/lifestyle and management of diabetes, something one cannot easily control in a patient population. Nevertheless, patients with diabetes presented with reduced intratumor 1,5-AG levels and an increase in food- and microbiome-derived metabolites, both consistent with the breast cancer xenograft data. Reduced 1,5-AG is a biomarker of glucose spikes and has been found to associate with a generally increased cancer mortality in a study of Japanese men (22). The accumulation of microbiome-derived metabolites that we observed in the tumor xenografts may contribute to the proinflammatory environment with increased ROS that has been described from syngeneic breast tumors in diabetic mice; however, none of these previous studies investigated the tumor metabolome that associates with diabetes. Several diabetes-associated metabolites — e.g., imidazole propionate, trimethylamine N-oxide, and phenyl sulfate — are formed by the gut microbiome and may increase oxidative stress in cells and inflammation in tumors (28, 29, 57–59).

Advanced glycation end products are sugar metabolites that build up in patients with diabetes. They are proinflammatory, mutagenic, and oncogenic, and their accumulation may raise breast cancer risk and mortality (32, 60–62). We detected an increased intratumor abundance of them under diabetic conditions and could show that one of the best-known advanced glycogen end products, CML, activates oncogenic and inflammatory pathways in breast cancer cells, in line with RAGE signaling. Thus, besides microbiome-derived metabolites, the increased exposure to advanced glycation end products may as well contribute to the more aggressive nature of breast cancer in patients with diabetes.

Oxidative stress promotes DNA strand breaks and genomic instability in cancer cells. Hyperglycemia increases ROS and DNA damage, as shown by our data and by others (38). These cells may also experience a loss in DNA damage repair capacity. A compromised DNA repair capacity has previously been linked to diabetes-induced fibrosis (63). We found that a deficiency in base excision repair, mismatch repair, and homologous recombination is strongly suggested by the gene expression profile and mutational signature in breast tumors from patients with diabetes, independent of the tumor ER status. Two mutational signatures that were present in these tumors may originate from a reduced DNA excision repair capacity, as suggested by the COSMIC signature compendium. Our observations from patient tumors were further confirmed by the transcriptome data obtained from our mouse xenograft model using diabetes-prone Akita mice and experimental data from high-glucose–cultured human breast cancer cell lines.

Homologous recombination is a key pathway in repairing double-stranded breaks that frequently occur under oxidative stress (64). Homologous recombination deficiency due to mutational inactivation of the *BRCA1* and *BRCA2* tumor suppressor genes is known to make human tumors susceptible to PARP inhibitors and platinum-based chemotherapies (65). PARP inhibitors have, therefore, been developed to treat patients with cancer with *BRCA*-inactivating mutations (66). We investigated whether human breast cancer cells, when cultured under hyperglycemia, are more vulnerable to DNA repair inhibitors, assessing a panel of cancer drugs that included the checkpoint kinase inhibitor AZD7762, the ATR (ataxia telangiectasia and Rad3 related) inhibitor berzosertib, the topoisomerase II inhibitor etoposide, and the PARP inhibitor olaparib. This line of experiments indicated a raised vulnerability of human breast cancer cells to these drugs under hyperglycemic conditions. We also showed that hyperglycemia downregulates DNA repair capacity, namely NHEJ, which is the primary pathway for repair of double-stranded breaks throughout the cell cycle, including the G2 phase, using a reporter assay. Based on these observations, we reason that diabetes-associated breast tumors may show an increased drug response to DNA damage repair inhibitors that are cancer therapeutics. We further argue that these observations should be followed up with clinical studies. Diabetes negatively and disproportionately affects underserved populations (20) and increases breast cancer mortality on a global scale. Any improvement in treating these patients with breast cancer should have a large effect.

As a strength of our study, we used a comprehensive approach to investigate the effects of diabetes and hyperglycemia on breast cancer biology and included tumors and cell lines from African American patients with breast cancer. However, patients with breast cancer with diabetes will use a variety of drugs that treat diabetes. The use of these drugs should make patients with diabetes more similar to patients without diabetes (i.e., dilute the signal of diabetes). It is a limitation of our study that we could not investigate how treatment of diabetes may have influenced our tumor data. We also could not associate duration of diabetes and circulating hemoglobin A1c levels with our tumor data, since these data were only available for a subset of the patients in our study. Another limitation of our study relates to the use of the Akita mouse as a diabetes model. The disease resembles type 1 diabetes but shows some phenotypes of type 2 diabetes (18, 19). These mice are immunocompromised but develop hyperglycemia with 100% penetrance in a well-defined age range, allowing tumor xenografts to grow in mice with diabetes with little experimental variation. It has been shown that hyperglycemia leads to immune function changes in breast tumors, using syngeneic mouse models (14). Despite these limitations, the transcriptome- and metabolome-based signatures in the human xenografts grown in diabetic Akita mice showed significant overlaps with the signatures detected in breast tumors of patients with diabetes. They point to the same consistent alterations across these lines of investigation, indicating identical diabetes/hyperglycemia-induced effects in them. These findings make us confident that our observations are valid, thereby supporting our study design. In addition, the key findings that hyperglycemia induces ROS and a condition of DNA repair deficiency were experimentally validated.

In summary, our investigation reveals an effect of diabetes on the biology of human breast tumors, largely independent of the tumor ER status, and provides a large data set of metabolome and transcriptome data as a resource for others to use in examination of the effects of diabetes in patient tumors. Diabetes may broadly activate developmental pathways that become oncogenic in the context of cancer and promote increased cell mobility and the odds of metastasis. Food- and microbiome-derived metabolites increase in these breast tumors, potentially affecting tumor biology by increasing diabetes-associated inflammation. Through an increase of ROS, diabetes also augments DNA damage in cancer cells while diminishing their abilities of repairing DNA lesions and strand breaks. Clinically, these events may lead to an increased drug response to DNA damage repair inhibitors among patients with diabetes-associated breast cancer.

## Methods

### Reagents.

All information is provided in Supplemental Methods.

### Collection of human breast tumors and patient data.

Patients with breast cancer undergoing surgery were recruited at the University of Maryland Medical Center. We previously described recruitment of this patient cohort (16, 67). Details are provided in Supplemental Methods.

### Orthotopic tumor growth in mice with diabetes/hyperglycemia.

Female Akita mice (NOD.Cg-Rag1^tm1Mom^ Ins2^Akita^ Il2rg^tm1Wjl^/SzJ) (68) were obtained from The Jackson Laboratory. This mouse strain is partly immunodeficient and develops hyperglycemia with a defined onset at 4 weeks of age. The animals are heterozygote carriers of a mutation in the *insulin 2* gene. The mutation induces misfolded protein and β cell death. Akita mice are a model of genetically induced hyperglycemia with similarities to type 1 diabetes but exhibit some phenotypes of type 2 diabetes (18, 19). We used the isogenic strain NOD.Cg-Rag1^tm1Mom^ Il2rg^tm1Wjl^/SzJ (69) as a matched control in this study. Further information and description of the orthotopic xenograft experimental protocol can be found in the Supplemental Methods.

### Cell lines.

All human breast cancer lines were obtained from American Type Culture Collection (ATCC). MDA-MB-157, MDA-MB-231, MDA-MB-436, and HCC1806 cells were grown in DMEM with 2 mM glutamine (MilliporeSigma) and 10% FBS. MDA-MB-175, MDA-MB-468, Hs578T, ZR-75-30, HCC1937, and HCC1500 cells were grown in RPMI media supplemented with 10% FBS. We obtained authentication of these cell lines through ATCC services, prior to and after completion of all experiments, using a short tandem repeat analysis. MDA-MB-231-LM2-mCMV-mcherry and MDA-MB-231-LM2-SORE6-mcherry cell lines were obtained from Lalage M. Wakefield (NCI, NIH, Bethesda, Maryland, USA). These cell lines were maintained in RPMI media supplemented with 10% FBS.

### Hyperglycemia in cell culture.

We performed in vitro experiments mimicking diabetes-like conditions by culturing breast cancer cells in high-glucose medium. We used a 5 mM glucose level as a control condition and 25 mM levels to model hyperglycemia (70). Both ER^+^ (MDA-MB-175, ZR-75-30, HCC1500) and ER^–^ (MDA-MB-157, MDA-MB-231, MDA-MB-468, Hs578T) human breast cancer cell lines were used for these experiments. Briefly, cells were plated in respective plates/flasks. The next day, the medium was removed, and cells were washed with PBS and then cultured with either low- (5 mM) or high-glucose (25 mM) media without serum. Prior to starting the high-glucose treatment experiments, we conditioned the cells with low-glucose medium and then switched to 25 mM glucose while we maintained the 5 mM glucose for the control cells. In some experiments, we added 20 mM mannitol to the 5 mM glucose control group to yield the osmolarity of added 25 mM glucose. However, we did not find differences in the investigated phenotypes between experiments with and without added 20 mM mannitol (Supplemental Figure 2, F and G).

### BrdU incorporation assay to assess inhibition of cell proliferation by DNA repair pathway inhibitors.

Cell proliferation was measured with the BrdU cell proliferation kit from MilliporeSigma. For details, see Supplemental Methods.

### Cell viability assay.

Numbers of viable cells were estimated using the CellTiter-Blue cell viability assay from Promega. For details, see Supplemental Methods.

### Migration and invasion assay.

Cell migration and invasion was monitored using the xCelligence System technology (Roche) for real-time monitoring of cell movement with an electronic cell sensor array, following manufacturer’s instructions. For more details, see Supplemental Methods.

### Measurement of mitochondrial ROS.

Mitochondrial superoxide in the breast cancer cells was measured following an earlier described method (71). For details, see Supplemental Methods.

### Immunofluorescence microscopy to quantify DNA damage.

Immunostaining of DNA damage markers (γH2A.X and 53BP1) was performed in MDA-MB-231 and Hs578T cells under hyperglycemic condition. Procedures are described in the Supplemental Methods.

### γH2AX IHC.

Accumulation of nuclear γH2AX protein was examined in 105 formalin-fixed and paraffin-embedded breast tumor tissues obtained from the Department of Pathology at the University of Maryland Clinical Center. Twenty-nine tumors (17 ER^+^ [59%], 11 ER^–^ [38%], 1 ER unknown) were obtained from patients with diabetes at time of disease diagnosis and 76 (45 ER^+^ [59%], 30 ER^–^ [39%], 1 ER unknown) from patients who were nondiabetic at disease diagnosis. We used the anti–phospho-histone H2A.X (Ser139) rabbit antibody from Cell Signaling (catalog 9718) at a 1:800 dilution to visualize γH2AX protein in the tumor sections. Nuclear γH2AX in the tumor epithelium was scored as negative, low, moderate, or high using a standard scoring system as previously described by us and others (72, 73). Scoring of the IHC was performed by a pathologist blinded to the diabetes status of the patients.

### Measurement of DNA repair capacity for NHEJ in breast cancer cells under hyperglycemic condition.

Assessment of the DNA repair capacity for NHEJ was carried out according to a previously described protocol (74), with some modifications. For details, see Supplemental Methods.

### Quantification of the mesenchymal phenotype in cell culture.

The quantification of mesenchymal morphology in Hs578T and MDA-MB-231 cells cultured under hyperglycemia is described in Supplemental Methods.

### Stemness reporter assay.

The assay has previously been described (37). For details, see Supplemental Methods.

### IC_50_/GI_50_ calculation using the GraphPad software.

Dose response measurements were analyzed using the GraphPad Prism software. The IC_50_ (alternatively defined as GI_50_ — as the concentration that results in inhibiting cell growth by 50%) for the various DNA repair pathway drugs under low- and high-glucose culture condition was calculated using nonlinear regression analysis (fitting a dose-response curve) in GraphPad prism 9. First, 100% (average absorbance of the control group) and “0% growth” (average absorbance blank from media without cell) were assigned in the growth curve. Then, the doses were log transformed, and absorbance values were normalized. The IC_50_ was calculated by nonlinear regression analysis (curve fitting) of normalized transformed data by selecting the model “absolute IC_50_ from normalized data”.

### Tumor proliferation score.

We selected a gene expression profile that included expression data for 11 cell cycle genes (*BIRC5*, *CCNB1*, *CDC20*, *CEP55*, *MKI67*, *NDC80*, *NUF2*, *PTTG1*, *RRM2*, *TYMS*, and *UBE2C*) and summed this profile into a metagene score as a marker for tissue proliferation, as described previously (16). The proliferation signature also contains *MKI67*, the transcript that encodes Ki67, a commonly used proliferation marker for tissues.

### Metabolome analysis of human tumors and xenografts.

The metabolome analysis of fresh-frozen human tumors and xenografts was performed using untargeted metabolic profiling by the service provider, Metabolon, Inc. For details, see Supplemental Methods.

### Transcriptome analysis of human tumors, xenografts, and cultured cells using RNA-Seq.

RNA was isolated from frozen breast tumors with and without diabetes (*n* = 36 diabetic and *n* = 37 nondiabetic) using the TRIzol method as described earlier (67). Isolation of RNA from the human xenografts (MDA-MB-231, MDA-MB-468 and HS578T with or without diabetes), breast cancer cells (MDA-MB-175, ZR7530, HCC1500, MDA-MB-157, MDA-MB-231, and MDA-MB-468) cultured under high glucose (25 mM), and MDA-MB-231 cells with or without CML was done using the RNeasy Plus Mini Kit (QIAGEN). Detailed information about the RNA-Seq methods, data analysis, and public access to the data is provided with the Supplemental Methods.

### Metabolomic and transcriptomic data integration.

For details, see Supplemental Methods.

### WES.

WES was performed by the service provider, Psomagen. Detailed information about the WES data generation, data analysis, and public access to the data is provided with the Supplemental Methods.

### GSEA and further validation using pathway “activity” scores.

GSEA and additional validation of the key pathways using activity scores are described in the Supplemental Methods.

### ssGSEA.

For details, see Supplemental Methods.

### IPA.

Genes commonly up- and downregulated in human breast tumors and xenografts (MDA-MB-231 and MDA-MB-468) comparing diabetic versus nondiabetic were uploaded into the IPA tool (*n* = 461 at FDR < 0.3 for differentially expressed genes) to analyze for their relationship with IPA pathways by calculating pathway enrichment scores. IPA maintains a large-scale pathway network derived from the Ingenuity Knowledge Base, which is a large, structured collection of observations in various experimental contexts with nearly 5 million findings manually curated from the biomedical literature or integrated from third-party databases. IPA calculates an enrichment score and *P* values using the Fisher’s exact test.

### Statistics.

All statistical tests were 2 tailed, and an association was considered statistically significant at *P* < 0.05. We used Student’s *t* test or Wilcoxon’s rank-sum test to assess the statistical significance of differences between 2 groups. We applied the 1-way ANOVA test to assess differences between more than 2 groups in order to determine whether the associated population means are significantly different. Exact *P* values for differences between 2 groups were obtained with post hoc tests. Statistical analyses were performed using Prism 8 (GraphPad), R software (https://www.r-project.org), the packages in Bioconductor provided by the R Foundation for Statistical Computing, or Qlucore Omics Explorer 3.7 (https://qlucore.com/omics-explorer).

### Study approval.

Collection of both biospecimens and the clinical and pathologic information was approved by the University of Maryland IRB (protocol no. 0298229). The research was also reviewed and approved by the NIH Office of Human Subjects Research Protections (OHSRP no. 2248). Informed written consent was obtained from all patients, and the research followed the ethical guidelines set by the Declaration of Helsinki.

### Data availability.

Metabolome data were deposited in the Open Science Framework (https://osf.io/h73rf/?view_only=). All RNA-Seq data generated for this study have been deposited in NCBI’s Gene Expression Omnibus database under the super series GSE202923, which is composed of subseries GSE202922, GSE202599, GSE202595, GSE202597, GSE202598, and GSE236420. The RNA-Seq data for the human breast tumors were deposited under accession no. GSE202922. The RNA-Seq data for the mouse xenografts were deposited under accession no. GSE202599. RNA-Seq data for the cell lines were deposited under accession nos. GSE202595 (ER^+^ cell lines), GSE202597 (CML-exposed MDA-MB-231 cells), GSE202598 (MDA-MB-157 cell line), and GSE236420 (MDA-MB-231 and MDA-MB-468 cell lines). The WES raw data for the breast tumors have been deposited in the Sequence Read Archive database under accession no. PRJNA840859.

All data values referred to in the main manuscript and supplemental materials, including the values for all data points shown in graphs and the values to support any reported means, are provided in the Supporting Data Values file.

## Author contributions

Study design was contributed by GP and SA. Supervision was contributed by SA. Animal experiments were contributed by GP. Cell culture experiments were contributed by GP, YO, and AA. Acquiring data was contributed by GP and YO. Analysis and interpretation of experimental data were contributed by GP. Computational analysis was contributed by GP, JC, WT, AZ, and TZM. Data visualization was contributed by GP and JC. Data resources, databases, patient recruitment, biospecimen collection and processing, and submission of data for public access were contributed by THD, JSB, TZM, AA, AC, DM, OI, XWW, HL, CAL, and AMN. Classification of human tumors was contributed by HGY. IHC was contributed by THD. Animal histology slide evaluation was contributed by ALF and YO. Writing of the original draft was contributed by GP and SA. Review and editing of the manuscript were contributed by GP, SA, and JC. Addressing of reviewer comments was contributed by GP and SA.

## Supplementary Material

Supplemental data

Supplemental data set 1

Supplemental data set 2

Supplemental data set 3

Supplemental table 1

Supplemental table 10

Supplemental table 2

Supplemental table 3

Supplemental table 4

Supplemental table 5

Supplemental table 6

Supplemental table 7

Supplemental table 8

Supplemental table 9

Supporting data values

## Version 1 10/31/2023

In-Press Preview

## Version 2 12/08/2023

Electronic publication

## Version 3 12/14/2023

Duplicated text was removed
