# Supplementary material for: Diabetes-associated breast cancer is molecularly distinct and shows a DNA damage repair deficiency
Source: JCI Insight. 2023 Dec 8;8(23):e170105. doi: 10.1172/jci.insight.170105 (PMC10795835; doi:10.1172/jci.insight.170105)
Supplement: Supplemental data [file jciinsight-8-170105-s180.pdf]

Supplemental Figures

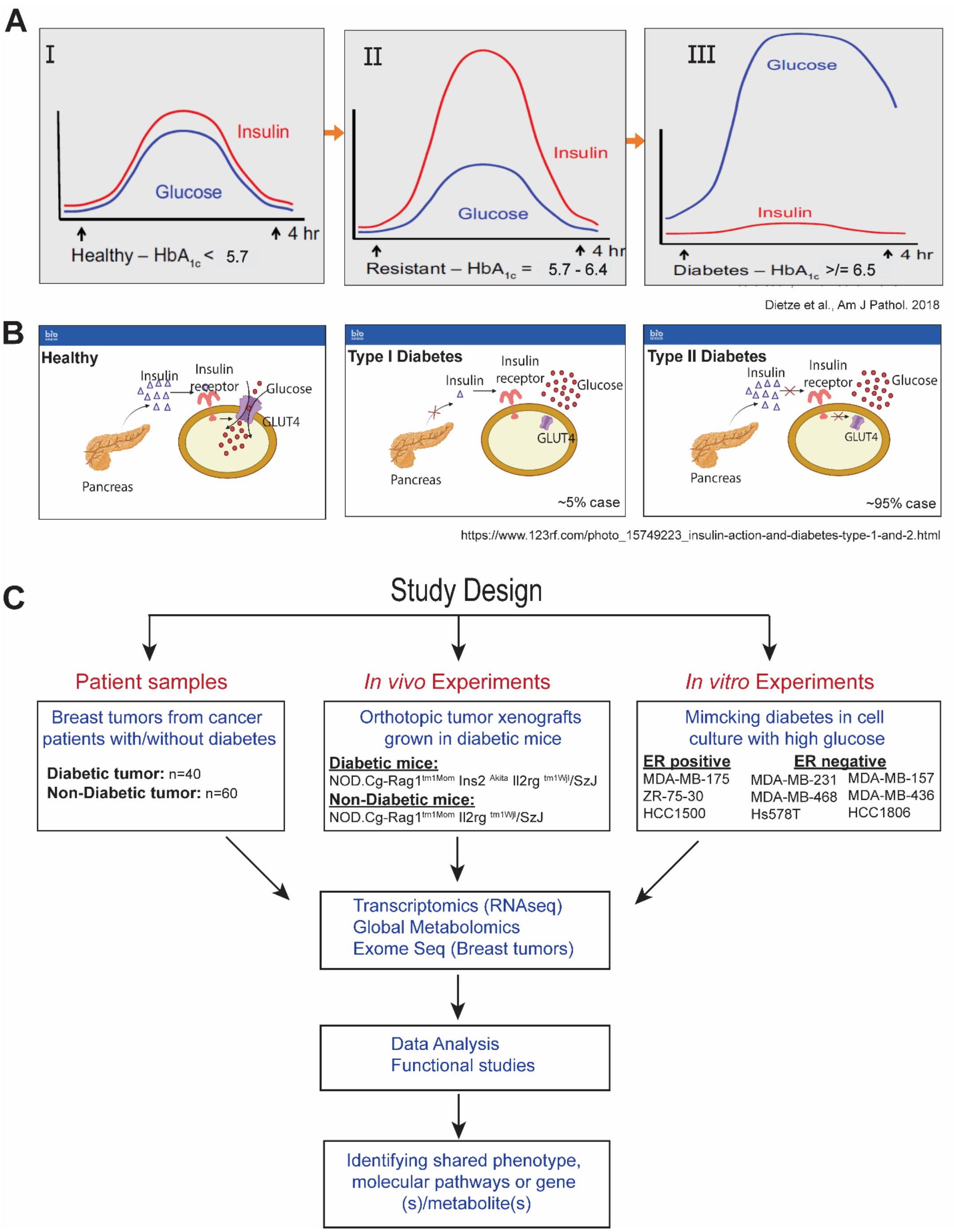

Fig. S1. Hallmarks of diabetes and design of our research study. (A) Glucose and insulin levels in healthy (I), pre-diabetic (II) and diabetic individuals (III). **(B)** Hyperglycemia is a key hallmark of both type I and type II diabetes. **(C)** Study design showing the three-pronged approach to investigate the impact of diabetes and hyperglycemia on breast cancer biology.

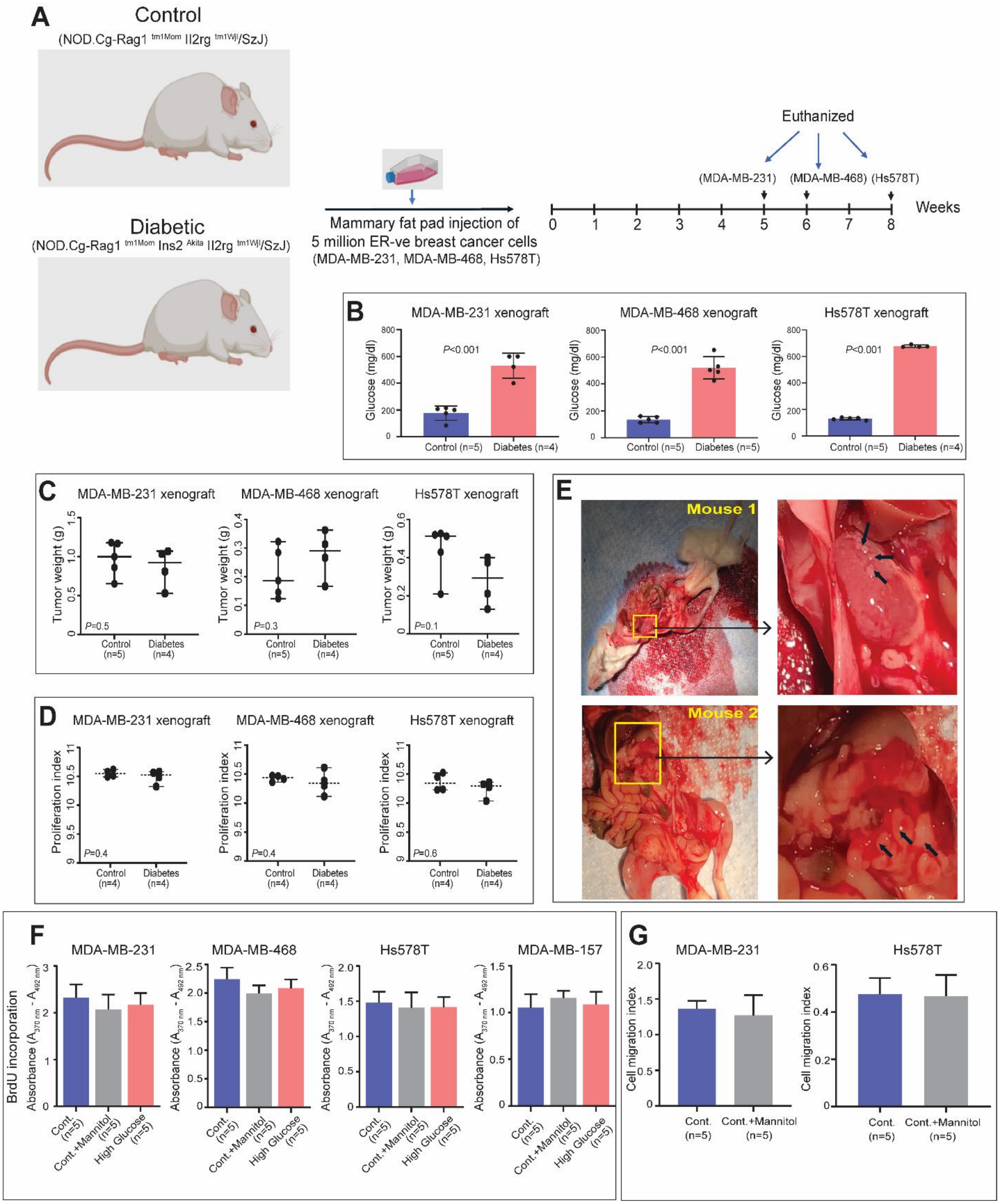

Fig. S2. Effect of hyperglycemia on growth and metastasis of orthotopic human xenografts and breast cancer cells cultured under hyperglycemia. (A) Experimental design of the mouse xenograft experiments using diabetes-prone Akita mice. **(B)** Measurement of blood glucose levels in mice prior to termination of the experiments. Data represent mean ± SD for n = 4-5; t-test was used for significance testing. **(C)** Tumor weights of MDA-MB-231, MDA-MB-468, and Hs578T xenografts collected from non-

diabetic (control) and diabetic mice. **(D)** Proliferation index of the MDA-MB-231, MDA-MB-468, and Hs578T xenografts collected from non-diabetic (control) and diabetic mice. Data represent mean ± SD for n = 4-5. t-test was used for significance testing. **(E)** Metastases to spleen, kidney, and upper gastrointestinal tract in MDA-MB-231 in two diabetic Akita mice. Arrows highlight metastatic lesions. **(F)** Proliferation of MDA-MB-231, MDA-MB-468, Hs578T and MDA-MB-157 cells under hyperglycemic conditions (25 mM glucose). Control experiments (5 mM glucose) were conducted with and without added 20 mM mannitol to adjust for osmolarity. A BrdU incorporation assay was used to measure proliferation 48 hours after plating. Data represents mean ± SD of 5 replicates in each group with t-test for significance testing. (**G**) Adding 20 mM mannitol to the culture medium of human breast cancer cells does not affect their migration pattern in the xCelligence-based assay system. Cells were cultured with 5 mM glucose.

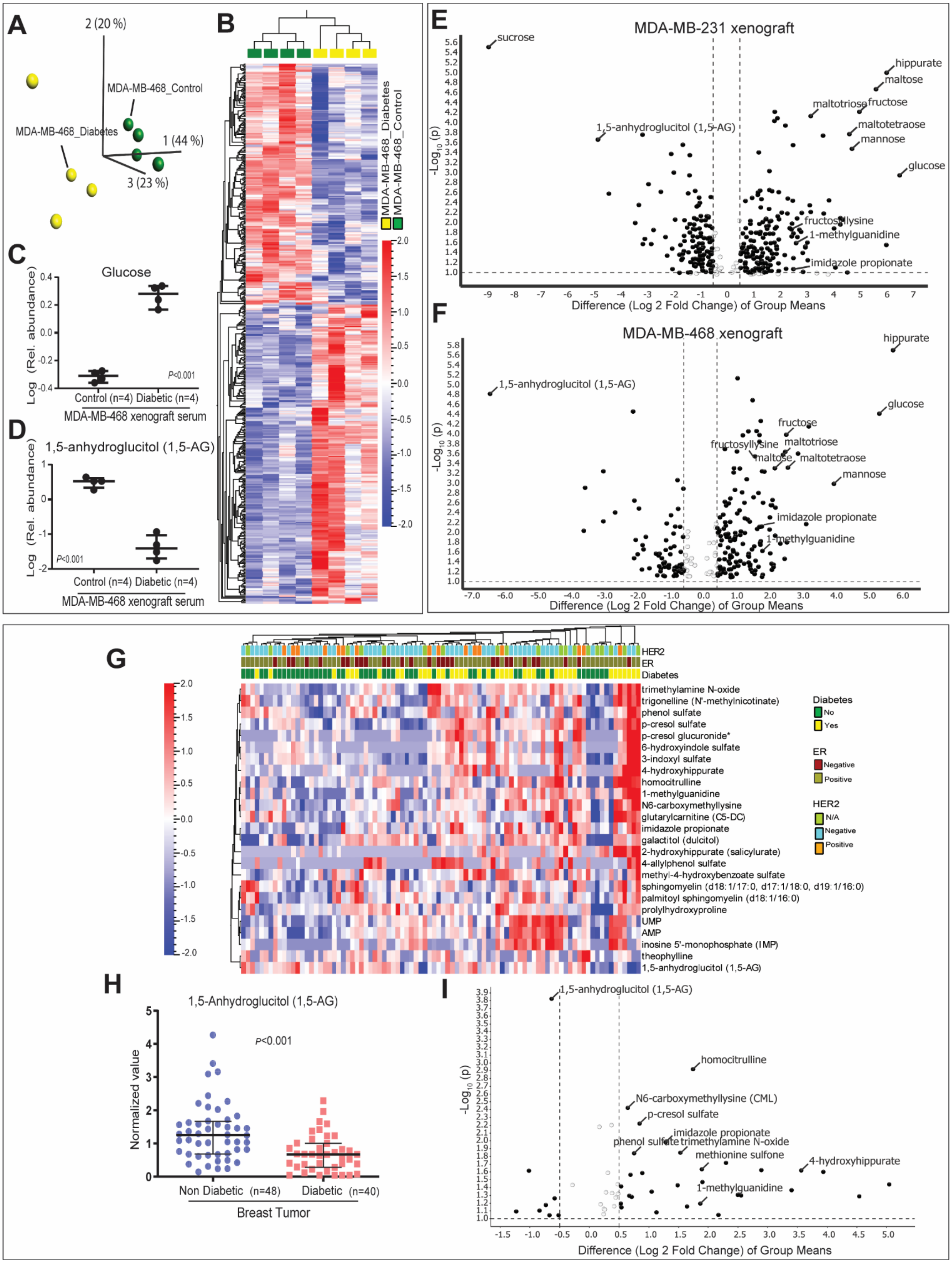
A
B
C
Glucose
Control (n=4) Diabetic (n=4)
MDA-MB-468 xenograft serum
D
1,5-anhydroglucitol (1,5-AG)
MDA-MB-468_Control
MDA-MB-468_Diabetes
E
MDA-MB-231 xenograft
Difference (Log 2 Fold Change) of Group Means
F
MDA-MB-468 xenograft
G
H
1,5-Anhydroglucitol (1,5-AG)
Non Diabetic (n=48)
Diabetic (n=40)
Breast Tumor
I

Fig. S3. Diabetes/hyperglycemia-induced metabolome signatures in tumor xenograft-bearing mice and breast tumors from patients with or without diabetes. (A-D) Metabolites in serum of diabetic and non-diabetic mice implanted with MDA-MB-468 xenografts. Serum samples were obtained when the tumor-bearing mice were euthanized to conclude the experiments 6 weeks after the orthotopic inoculation of the MDA-MB-468 cells. **(A)** Unsupervised PCA using the serum metabolite data obtained from the non-diabetic (control) and diabetic mice. The plot shows a separation based on the diabetes status of the mice. **(B)** Heatmap emphasizing the difference in serum metabolite levels between non-diabetic and diabetic mice (FDR cutoff < 0.3 for inclusion of metabolites). **(C,D)** Serum level of the diabetic markers, glucose and 1,5 anhydroglucitol (1,5-AG), in the MDA-MB-468 xenografts by diabetes status. Data represent mean ± SD of log transformed relative abundance levels (n=4 each group). t-test for significance testing. **(E,F)** Volcano plots to highlight differences in intratumor metabolite levels between diabetic and non-diabetic mice bearing tumor xenografts. **(E)** Volcano plot showing differences in intratumor metabolite levels ranked by both group means (x-axis: positive for metabolites upregulated and negative for metabolites downregulated in diabetic mice) and significance level (y-axis: P value) for the observed difference between diabetic and non-diabetic mice bearing MDA-MB-231 tumor xenografts. Cutoff for inclusion of metabolites: $P < 0.05$ and fold change ≥ 1.5. (**F)** Same volcano plot but for differences between diabetic and non-diabetic mice bearing MDA-MB-468 tumor xenografts. Cutoff for inclusion of metabolites: $P < 0.05$ and fold change ≥ 1.5. Intratumor 1,5-AG is consistently downregulated, and glucose is upregulated in the Akita mice with diabetes. Several key food- and microbial-derived metabolites are highlighted. **(G-I)** Metabolite profiles in breast tumors of patients with (n=40) or without (n=48) diabetes. **(G)** Heatmap highlighting intratumor metabolite differences between diabetic and non-diabetic patients ($P < 0.05$ cutoff for inclusion). **(H)** Intratumor level of the diabetic marker, 1,5 AG, in breast tumors from non-diabetic and diabetic patients. Data represent median with interquartile range. Mann Whitney U test was used for significance testing. **(I)** Volcano plot showing differences in intratumor metabolite levels ranked by both group means (positive for metabolites upregulated and negative for metabolites downregulated in diabetic patients) and significance level for the observed difference between diabetic and non-diabetic patients.

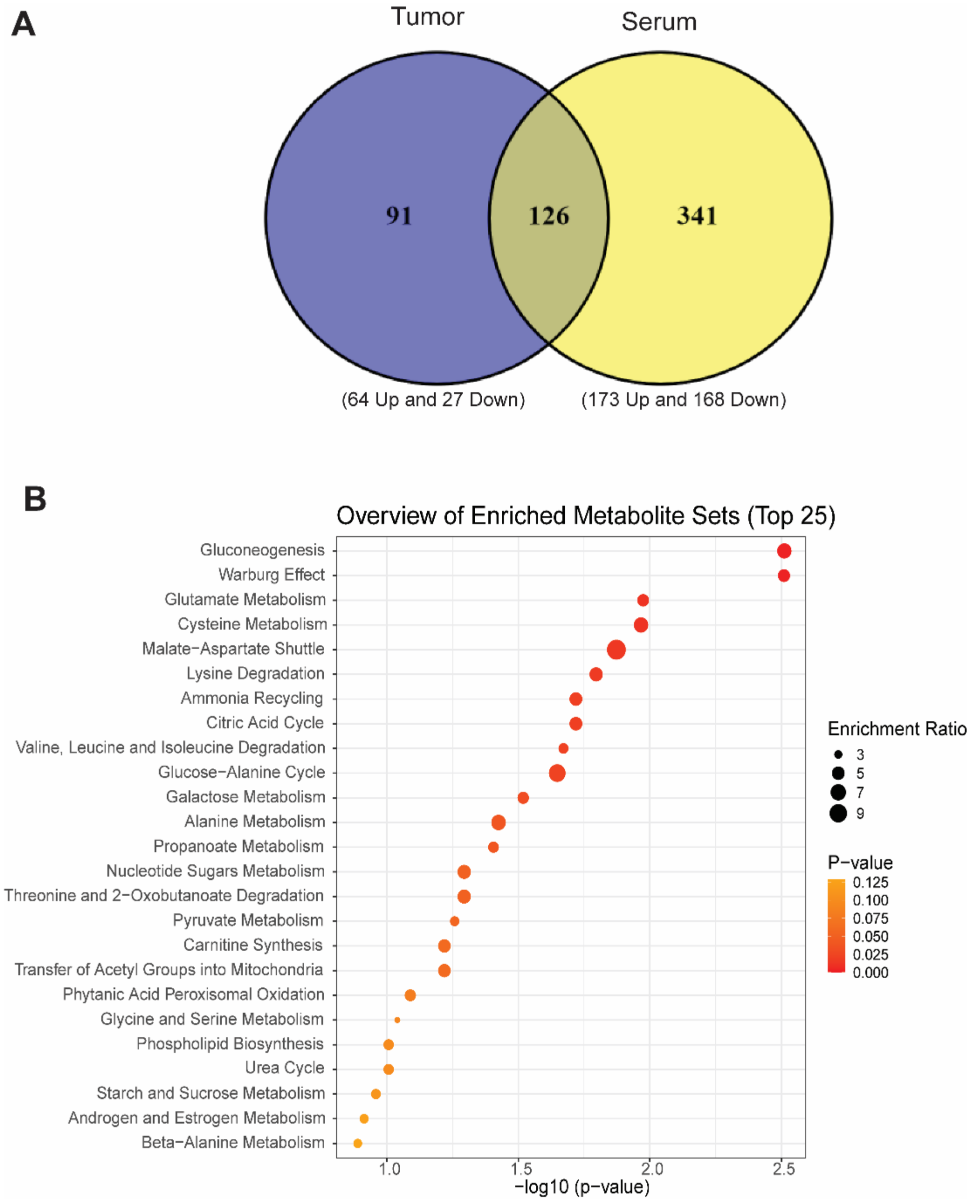

Fig. S4. Diabetes-associated metabolites in tumors and sera of mice harboring MDA-MB-468 tumor xenografts. (A) Venn diagram of diabetes-associated metabolites in either tumors, sera, or both, of mice harboring MDA-MB-468 tumor xenografts. Included metabolites are those with a difference between diabetic and non-diabetic mice in either tumors, sera, or both, at FDR < 0.3. Ninety-one diabetes-associated metabolites (64 up and 27 down) are distinctively present in tumors and 341 (173 up and 168 down) in serum samples. **(B)** Pathway enrichment analyses in MetaboAnalyst with the 91 diabetes-associated metabolites distinctively altered in tumors. Gluconeogenesis and the Warburg effect are the top enriched pathways.

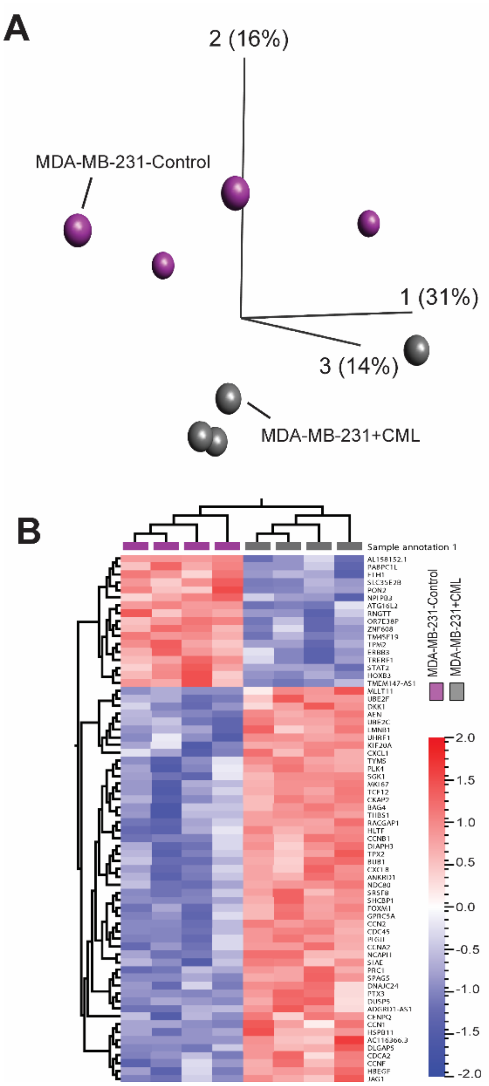

C HALLMARK GSEA Analysis

Name	NES	q
HALLMARK_TNFA_SIGNALING_VIA_NFKB	1.62298	0.01984
HALLMARK_MITOTIC_SPINDLE	1.62297	0.02133
HALLMARK_E2F_TARGETS	1.71051	0.02579
HALLMARK_G2M_CHECKPOINT	1.67253	0.02579
HALLMARK_HYPOXIA	1.5936	0.03729
HALLMARK_MYC_TARGETS_V1	1.57767	0.03769
HALLMARK_ESTROGEN_RESPONSE_LATE	1.56469	0.03797
HALLMARK_MTORC1_SIGNALING	1.54595	0.03989
HALLMARK_ALLOGRAFT_REJECTION	1.54656	0.04166
HALLMARK_MYC_TARGETS_V2	1.51703	0.04344
HALLMARK_TGF_BETA_SIGNALING	1.50051	0.04797
HALLMARK_REACTIVE_OXYGEN_SPECIES_PATHWAY	1.49293	0.04893
HALLMARK_APOPTOSIS	1.48655	0.05112
HALLMARK_DNA_REPAIR	1.47935	0.05342
HALLMARK_INFLAMMATORY_RESPONSE	1.43162	0.07873
HALLMARK_EPITHELIAL_MESENCHYMAL_TRANSITION	1.43351	0.08226
HALLMARK_CHOLESTEROL_HOMEOSTASIS	1.41623	0.08682
HALLMARK_COMPLEMENT	1.39464	0.11384
HALLMARK_UV_RESPONSE_DN	1.37723	0.12351
HALLMARK_ADIPOGENESIS	1.37175	0.13241
HALLMARK_ANGIOGENESIS	1.36563	0.13839
HALLMARK_FATTY_ACID_METABOLISM	1.36338	0.13958
HALLMARK_KRAS_SIGNALING_UP	1.35118	0.1449
HALLMARK_ANDROGEN_RESPONSE	1.34276	0.15564
HALLMARK_GLYCOLYSIS	1.31393	0.18075
HALLMARK_SPERMATOGENESIS	1.31397	0.18774
HALLMARK_ESTROGEN_RESPONSE_EARLY	1.30556	0.18941
HALLMARK_INTERFERON_GAMMA_RESPONSE	1.29546	0.20007
HALLMARK_APICAL_SURFACE	1.26966	0.23073
HALLMARK_KRAS_SIGNALING_DN	1.2473	0.23461
HALLMARK_IL2_STAT5_SIGNALING	1.24958	0.23967
HALLMARK_PROTEIN_SECRETION	1.26255	0.24116
HALLMARK_COAGULATION	1.25274	0.24456
HALLMARK_IL6_JAK_STAT3_SIGNALING	1.25574	0.2456

Fig. S5. Carboxymethyllysine (CML)-induced changes to the transcriptome in MDA-MB-231 cells. (A) MDA-MB-231 cells were treated with 1 µM CML for 48 hours. The unsupervised PCA plot shows a separation of control and CML-exposed MDA-MB-231 cells based on the whole cell transcriptome (n=4 for each group). **(B)** Heatmap of the metabolite differences between control and CML-exposed cells (FDR < 0.3 for inclusion of transcripts). **(C)** Enrichment of differently expressed genes in GSEA Hallmark gene sets ranked by FDR (q-value). NES, normalized enrichment score.

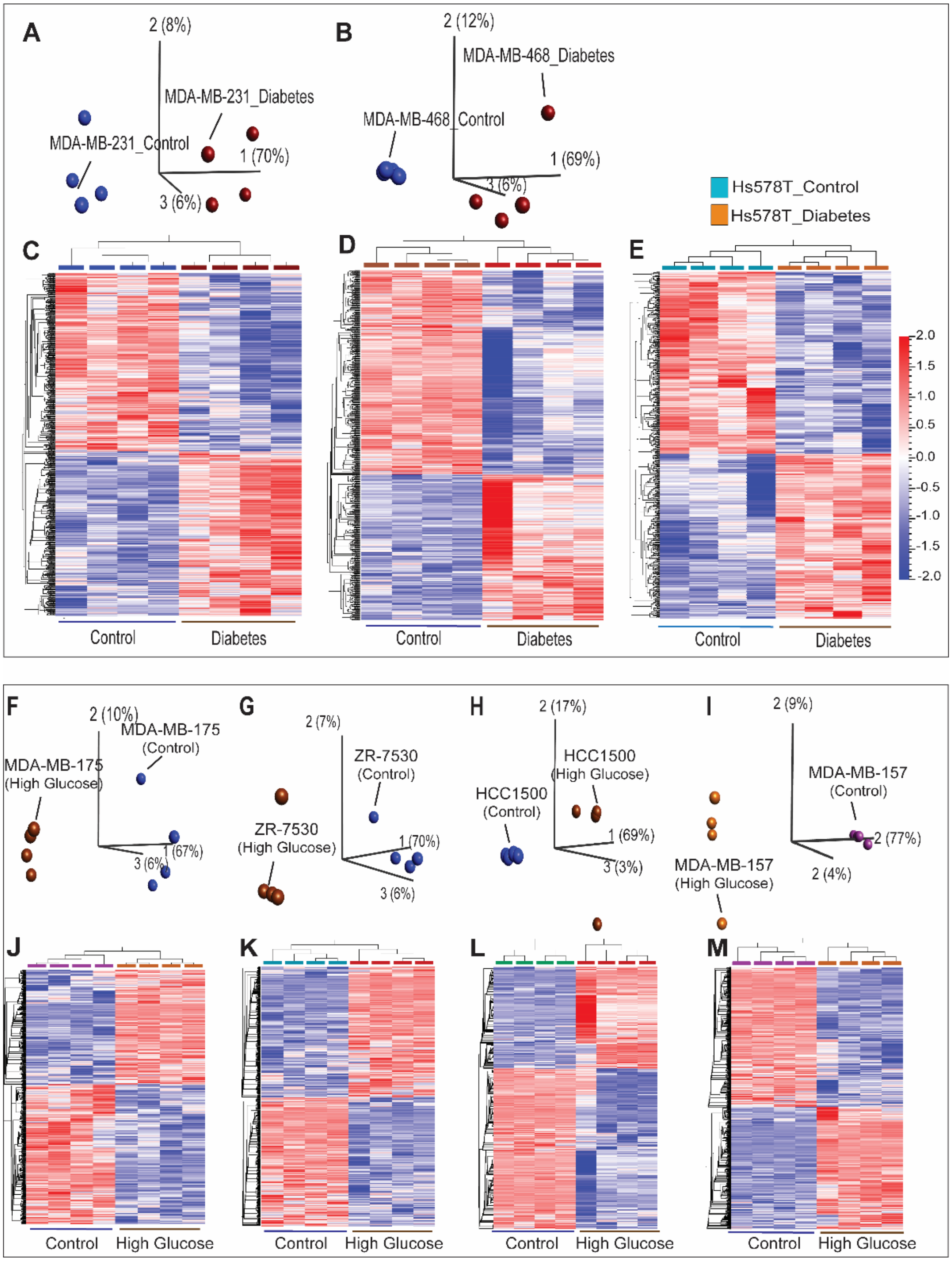

Fig. S6. Hyperglycemia induces robust changes in the transcriptome of tumor xenografts and cultured breast cancer cells. PCA plots show separation of MDA-MB-231 **(A)** and MDA-MB-468 **(B)** tumor xenografts by diabetes status using transcriptome data (FDR < 0.3 cutoff for inclusion of differentially expressed transcripts). Heatmaps highlighting the difference in gene expression by diabetes status for MDA-MB-231 **(C)**, MDA-MB-468 **(D)**, and Hs578T **(E)** xenografts. MDA-MB-231 and MDA-MB-468 xenografts: FDR < 0.3 as cutoff for inclusion of differently expressed transcripts. Hs578T: $P < 0.05$. **(F-M)** Gene expression patterns in breast cancer cells cultured under hyperglycemia. Unsupervised PCA plots

show separation of control (5 mM glucose) and high glucose (25 mM glucose = hyperglycemia)-cultured MDA-MB-175 **(F)**, ZR-75-30 **(G)**, HCC1500 cells **(H)**, and MDA-MB-157 cells **(I)** based on the whole cell transcriptome (n=4 for each group). (**J-M**) Heatmaps for the same comparisons with FDR < 0.3 as cutoff for inclusion of differentially expressed genes.

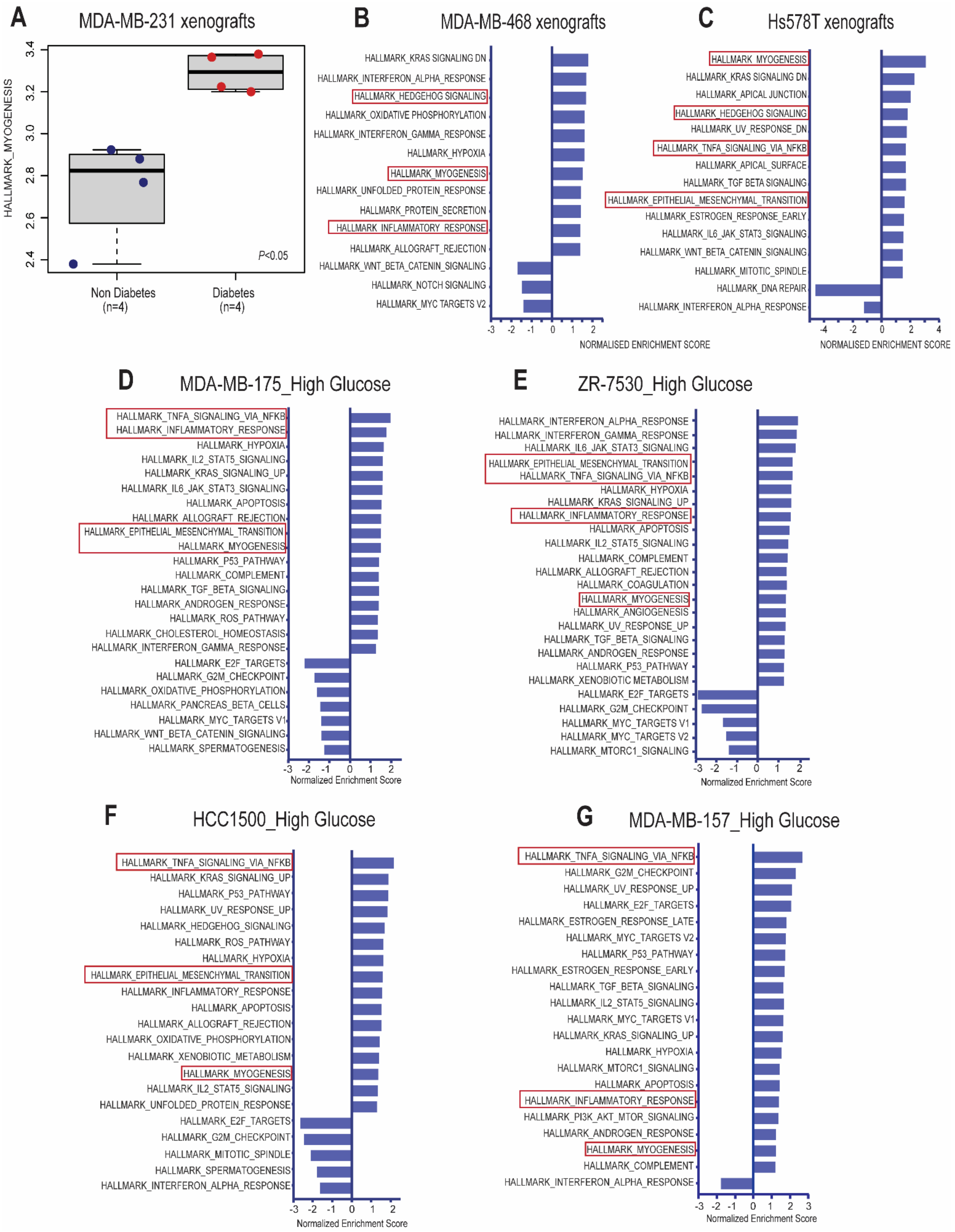
A
MDA-MB-231 xenografts
HALLMARK_MYOGENESIS
P<0.05
Non Diabetes (n=4)
Diabetes (n=4)
B
MDA-MB-468 xenografts
C
Hs578T xenografts
NORMALISED ENRICHMENT SCORE
D
MDA-MB-175_High Glucose
E
ZR-7530_High Glucose
F
HCC1500_High Glucose
G
MDA-MB-157_High Glucose
Normalized Enrichment Score

Fig. S7. GSEA using transcriptome data from either tumor xenografts grown in diabetic Akita mice or human breast cancer cell lines cultured under hyperglycemia. (A-C) Myogenesis signaling is upregulated in tumor xenografts of diabetic Akita mice. **(A)** Myogenesis signaling scores in MDA-MB-231 xenografts by diabetes status; Wilcoxon rank sum test for significance testing. Enriched GSEA Hallmark gene sets (comparing diabetic vs non-diabetic mice, FDR < 0.25) for MDA-MB-468 xenografts **(B)** and Hs578T xenografts **(C),** pointing to positive enrichment of differentially expressed genes in the myogenesis and hedgehog signaling pathways in presence of diabetes. Y-axis represents the enriched gene sets (either positive or negative) and X-axis represents the normalized enrichment scores (NES) for each gene set. **(D-G)** Enrichment of differentially expressed genes (hyperglycemia vs. control) in GSEA Hallmark gene sets (FDR < 0.25) for four human breast cancer cell lines, MDA-MB-175 **(D)**, ZR-75-30 **(E)**, HCC1500 **(F)**, and MDA-MB-157 **(G)**. Y-axis represents the enriched gene sets (either positive or negative), and X-axis represents the normalized enrichment scores (NES) for each gene set. Myogenesis is commonly enriched for differentially expressed genes, and so is EMT, NFκB-mediated TNFα signaling, and the inflammatory response, indicating increased signaling under hyperglycemia.

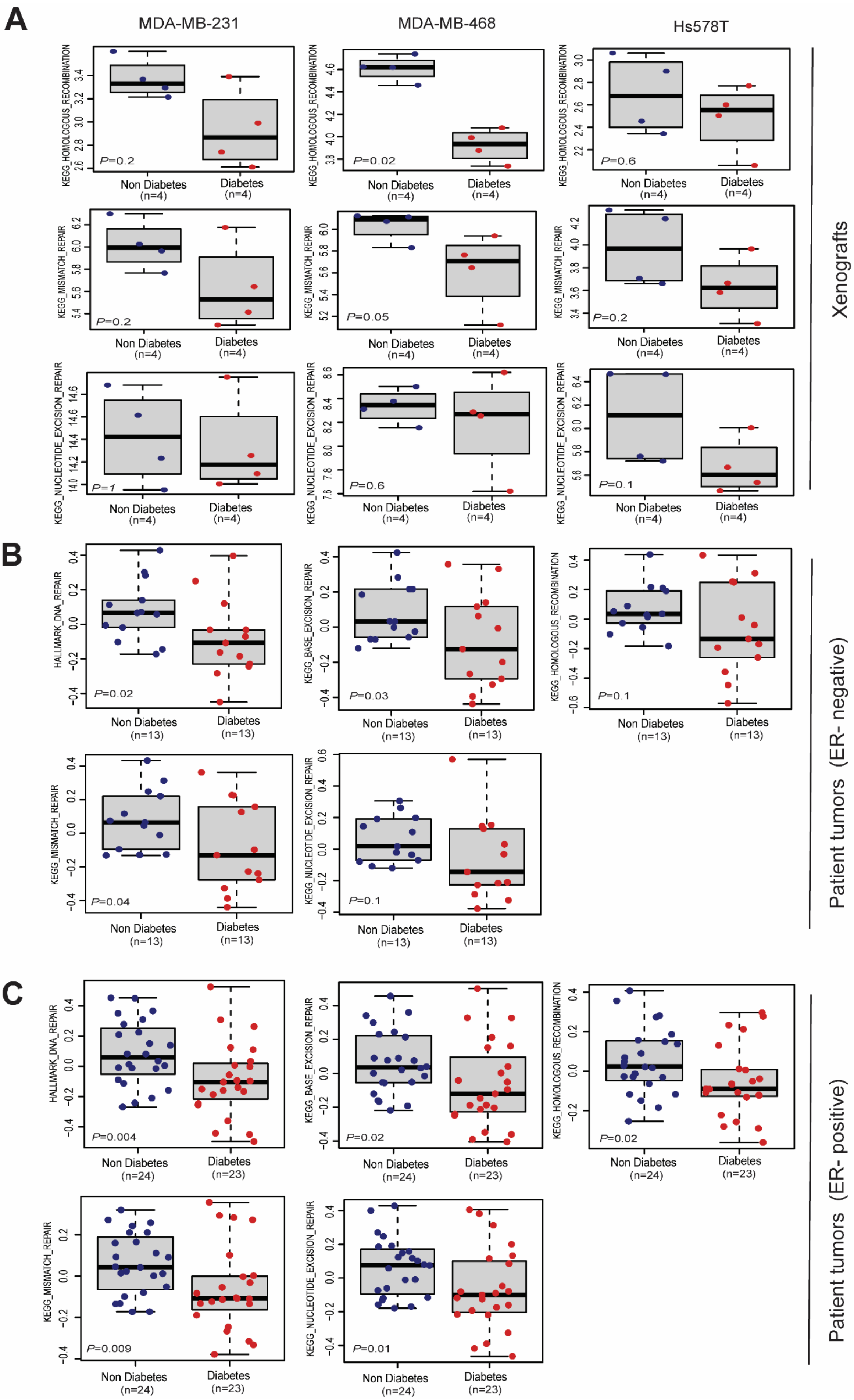
A
MDA-MB-231
MDA-MB-468
Hs578T
KEGG_HOMOLOGOUS_RECOMBINATION
KEGG_MISMATCH_REPAIR
KEGG_NUCLEOTIDE_EXCISION_REPAIR
P=0.2
P=0.02
P=0.6
P=0.2
P=0.05
P=0.2
P=1
P=0.6
P=0.1
Non Diabetes (n=4)
Diabetes (n=4)
Xenografts
B
HALLMARK_DNA_REPAIR
KEGG_BASE_EXCISION_REPAIR
KEGG_HOMOLOGOUS_RECOMBINATION
KEGG_MISMATCH_REPAIR
KEGG_NUCLEOTIDE_EXCISION_REPAIR
P=0.02
P=0.03
P=0.1
P=0.04
P=0.1
Non Diabetes (n=13)
Diabetes (n=13)
Patient tumors (ER- negative)
C
HALLMARK_DNA_REPAIR
KEGG_BASE_EXCISION_REPAIR
KEGG_HOMOLOGOUS_RECOMBINATION
KEGG_MISMATCH_REPAIR
KEGG_NUCLEOTIDE_EXCISION_REPAIR
P=0.004
P=0.02
P=0.02
P=0.009
P=0.01
Non Diabetes (n=24)
Diabetes (n=23)
Patient tumors (ER- positive)

Fig. S8. Down-regulation of DNA repair pathway capacity in xenografts and patient tumors in presence of diabetes. (A) Multiple DNA repair pathways are downregulated in tumor xenografts of diabetic Akita mice. Shown are KEGG-annotated DNA repair pathway scores for homologous recombination, mismatch repair and nucleotide excision repair in MDA-MB-231, MDA-MB-468 and Hs578T xenografts by diabetes status. Wilcoxon rank sum test for significance testing. **(B,C)** Down-regulation of DNA repair pathway activities in ER-negative and ER-positive breast tumors from patients with diabetes. **(B)** ER-negative breast tumors, **(C)** ER-positive breast tumors. Pathway scores are ssGSEA-based and covariate-adjusted for patient samples. The significance of the diabetes status controlled for covariates was assessed via multivariable linear regression.

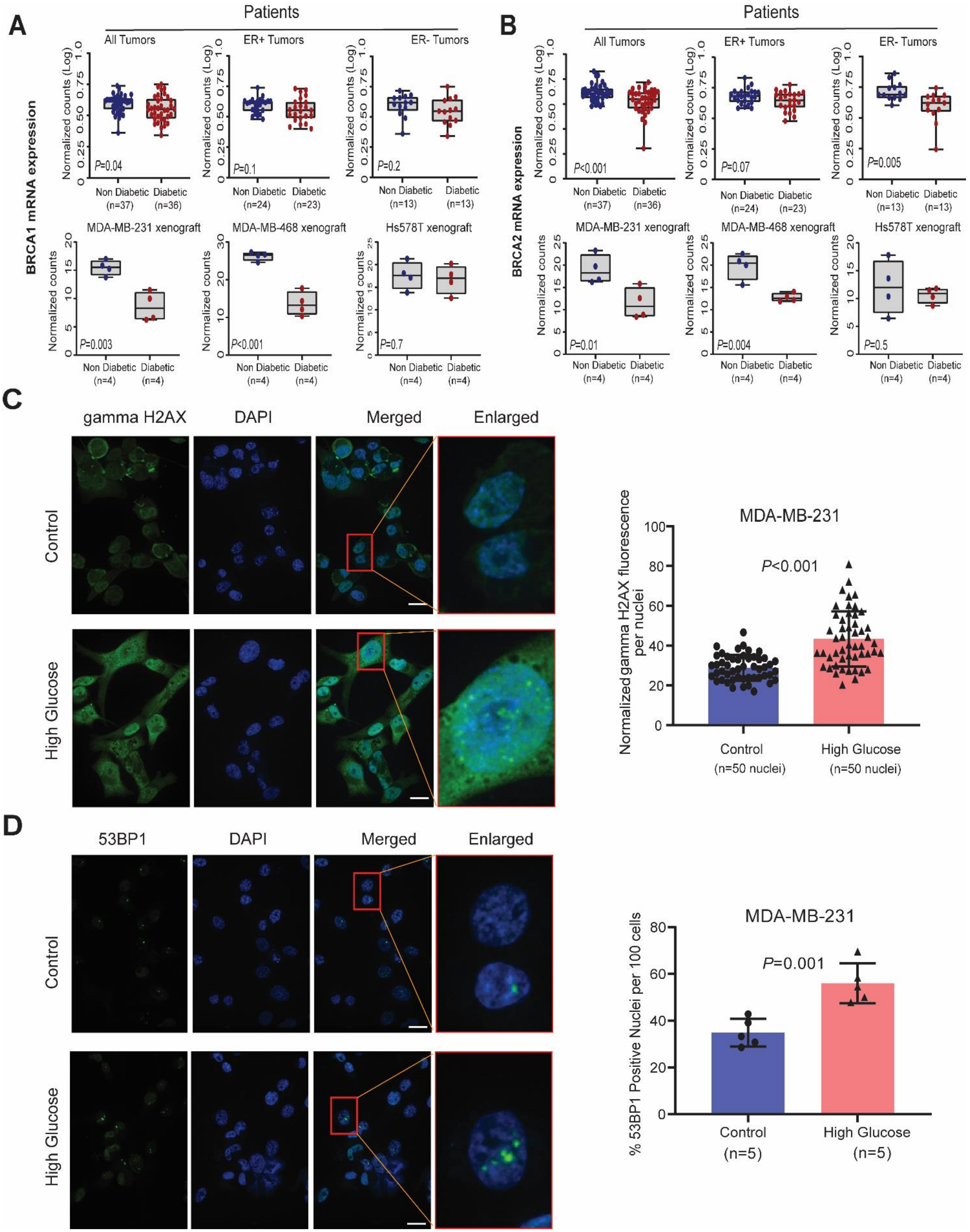
A
Patients
All Tumors
ER+ Tumors
ER- Tumors
BRCA1 mRNA expression
Normalized counts (Log)
P=0.04
P=0.1
P=0.2
Non Diabetic (n=37)
Diabetic (n=36)
Non Diabetic (n=24)
Diabetic (n=23)
Non Diabetic (n=13)
Diabetic (n=13)
MDA-MB-231 xenograft
MDA-MB-468 xenograft
Hs578T xenograft
Normalized counts
P=0.003
P<0.001
P=0.7
Non Diabetic (n=4)
Diabetic (n=4)
B
Patients
All Tumors
ER+ Tumors
ER- Tumors
BRCA2 mRNA expression
Normalized counts (Log)
P<0.001
P=0.07
P=0.005
Non Diabetic (n=37)
Diabetic (n=36)
Non Diabetic (n=24)
Diabetic (n=23)
Non Diabetic (n=13)
Diabetic (n=13)
MDA-MB-231 xenograft
MDA-MB-468 xenograft
Hs578T xenograft
Normalized counts
P=0.01
P=0.004
P=0.5
Non Diabetic (n=4)
Diabetic (n=4)
C
gamma H2AX
DAPI
Merged
Enlarged
Control
High Glucose
MDA-MB-231
Normalized gamma H2AX fluorescence per nuclei
P<0.001
Control (n=50 nuclei)
High Glucose (n=50 nuclei)
D
53BP1
DAPI
Merged
Enlarged
Control
High Glucose
MDA-MB-231
% 53BP1 Positive Nuclei per 100 cells
P=0.001
Control (n=5)
High Glucose (n=5)

Fig. S9. Down-regulation of BRCA1/2 in diabetic patients and mice and increase in DNA damage under hyperglycemia. (A-B) *BRCA1 & 2* transcript expression in patient tumors and tumor xenografts by diabetes status. Expression of *BRCA1* (**A**) and *BRCA2* (**B**) mRNA in tumors tends to be downregulated in presence of diabetes; t test for statistical analysis. **(C-D)** Hyperglycemia induces DNA damage in MDA-MB-231 cells. **(C)** Representative immunofluorescence images of γH2AX staining in MDA-MB-231 cells under hyperglycemia. Quantification of γH2AX in MDA-MB-231 cells comparing hyperglycemia (25 mM glucose vs. control with 5 mM glucose) using ImageJ software. Scale bar is 20 µm for γH2AX, DAPI and merged images. Data show mean ± SD of normalized fluorescence in 50 nuclei taken from five different representative areas for each group; t-test for significance testing. **(D)** Representative immunofluorescence images of 53BP1 staining in MDA-MB-231 cells under hyperglycemia. Scale bar is 20 µm for 53BP1, DAPI and merged images. Quantification of 53BP1 in MDA-MB-231 cells comparing hyperglycemia vs. control using ImageJ software. Data represent the mean ± SD of the average percentage of localized 53BP1 expression in positive nuclei in each group, using n=5 images from each group. t-test was used for statistical analysis.

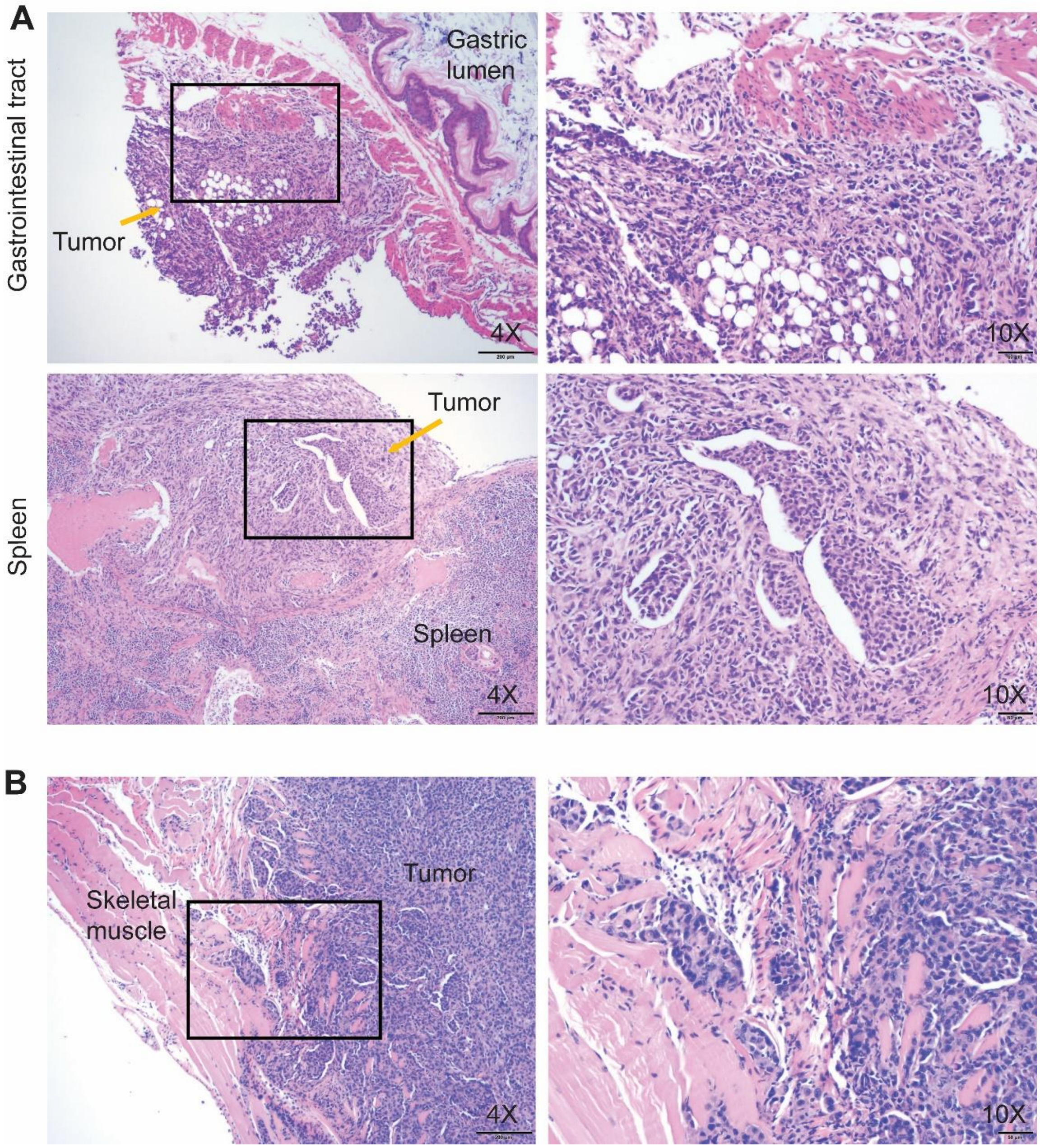

Fig. S10. Diabetes induces gastrointestinal tract and spleen metastasis and skeletal muscle invasion in the MDA-MB-231 breast cancer xenograft model. (A) Representative histological images (H&E staining) for metastatic nodules found in the gastrointestinal tract (top panel) and spleen (bottom panel) of hyperglycemic/diabetic Akita mice bearing MDA-MB-231 tumor xenografts. (**B)** Representative histological images (H&E staining) for skeletal muscle invasion in hyperglycemic/diabetic Akita mice bearing MDA-MB-231 tumor xenografts. Based on the gross examination, tumors either invaded the superficial, thin cutaneous muscle (panniculus carnosus) or invaded the abdominal skeletal muscles deep to the mammary fat pad.

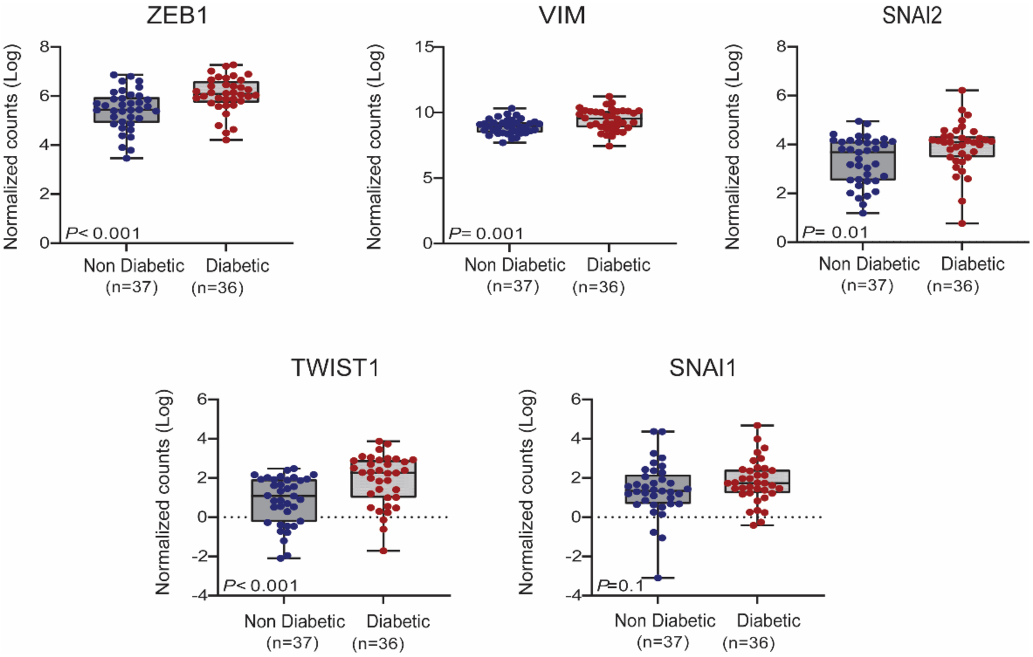

Fig. S11. Transcript expression of EMT driver genes in breast tumors comparing diabetic with non-diabetic breast cancer patients.

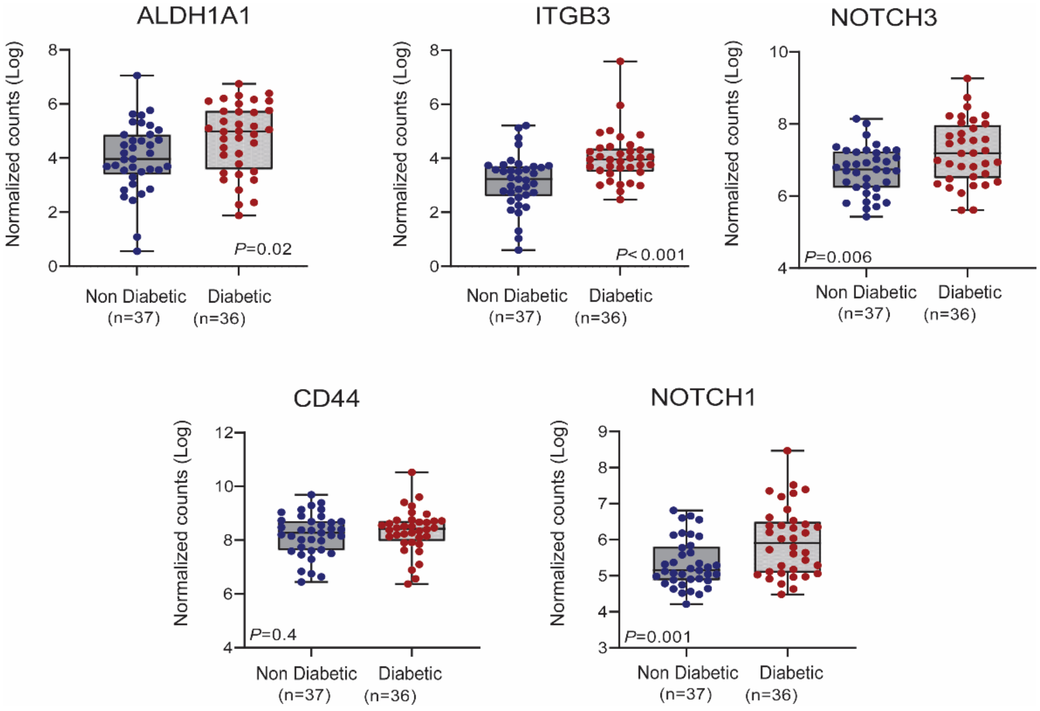

Fig. S12. Hyperglycemia activates a stem cell phenotype. Transcript expression of stem cell markers in breast tumors comparing diabetic with non-diabetic breast cancer patients.

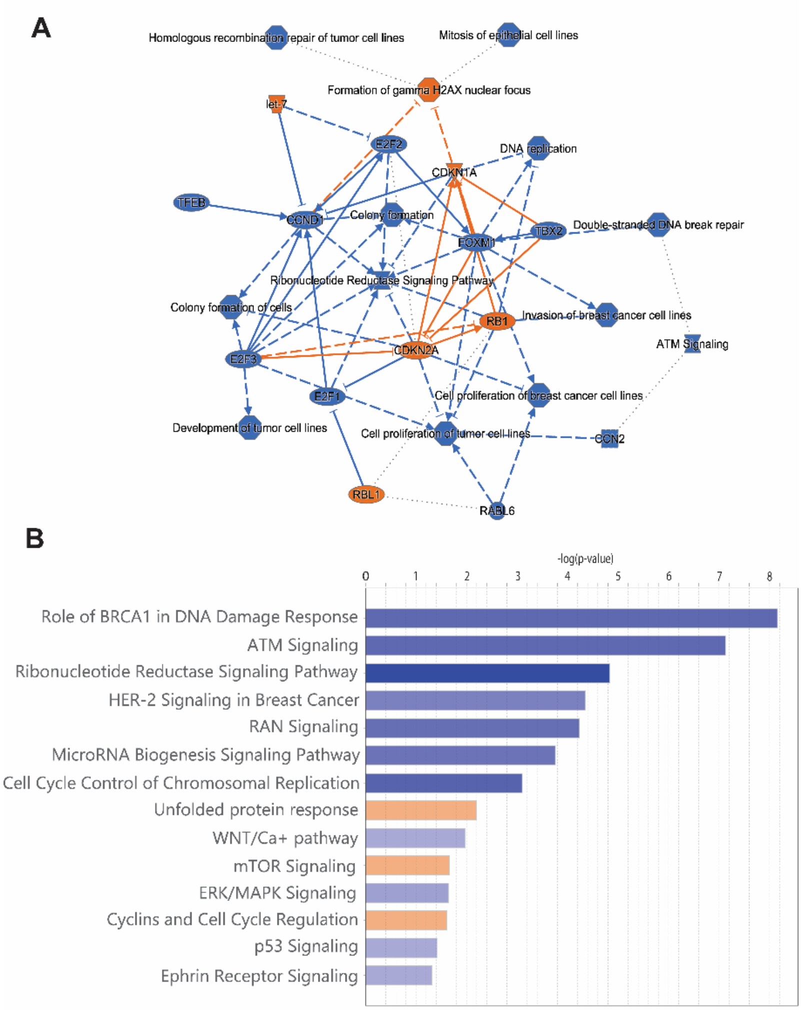

Fig. S13. Diabetes and hyperglycemia impair DNA repair capacity both in xenografts and breast cancer cell lines. (A-B) IPA analysis with 311 genes whose expression is commonly altered by diabetes/hyperglycemia in both xenografts (MDA-MB-231 and MDA-MB-468) and cell lines (MDA-MB-231 and MDA-MB-468) exposed to hyperglycemia (25 mM glucose). **(A)** Summary graph of the IPA analysis indicates activation of DNA damage signaling like “Formation of gamma H2AX nuclear focus” in presence of diabetes. Blue color indicates “inhibition” and red color indicate “activation” of a process. **(B)** Pathway enrichment analyses in IPA. Blue color indicates “inhibition” and red color indicate “activation” of a pathway/process by diabetes. “Role of BRCA1 in DNA damage response” is the top pathway indicated to be inhibited by diabetes.

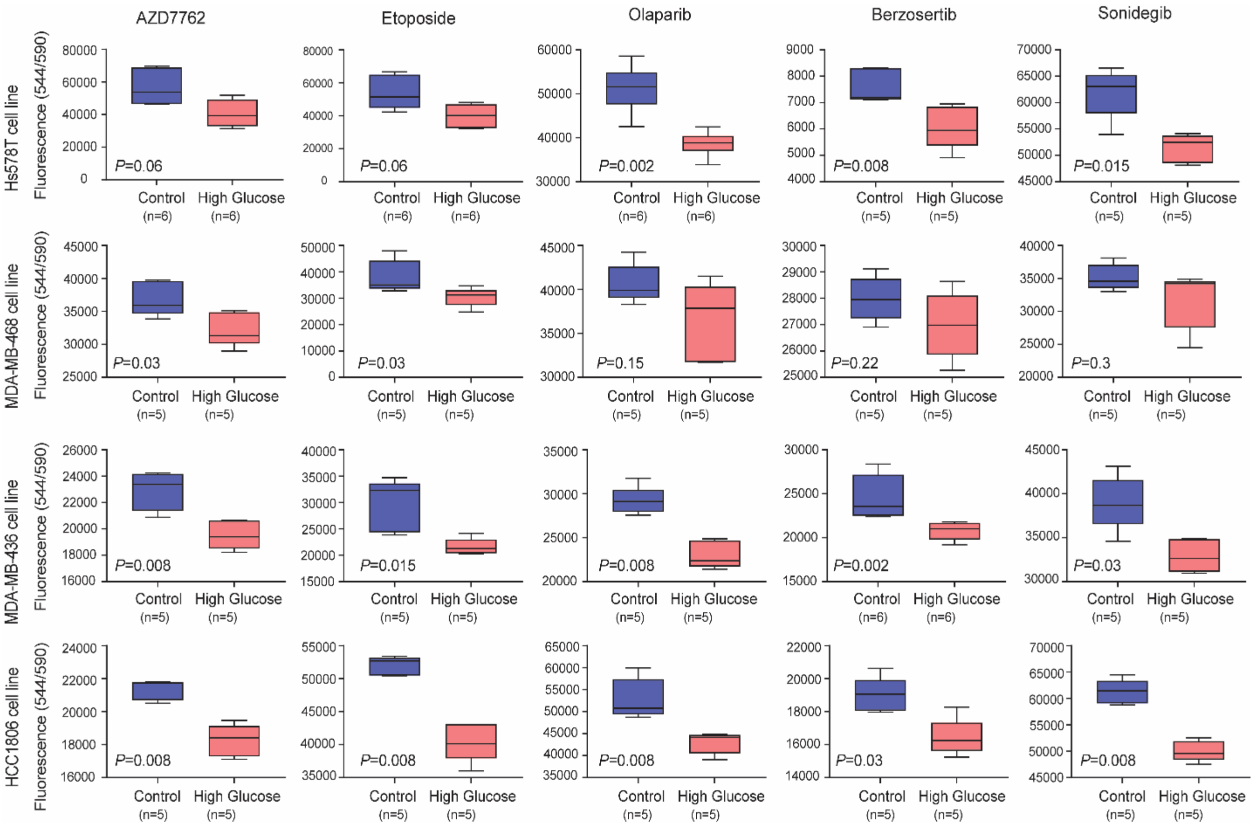

Fig. S14. Hyperglycemia increases sensitivity to drugs targeting the DNA damage repair pathway. Increased sensitivity of breast cancer cells to DNA damage repair and Hedgehog signaling (sonidegib) inhibitors under hyperglycemia. Decrease in viable cells of Hs578T, MDA-MB-468, MDA-MB-436 and HCC1806 cells following treatment with DNA repair pathway drugs e.g. AZD7762 (10 nM), etoposide (100 nM), olaparib (1 µM), berzosertib (1 µM), and sonidegib (100 nM) under hyperglycemia (25 mM glucose). Cell titer blue assay measuring the number of viable cells 48 hours after adding the inhibitors to the cell culture medium. Data represents mean ± SD with 5 replicates for each experimental group (control: 5 mM glucose). Wilcoxon rank sum test was applied for significance testing.

Supplemental Materials and Methods

Reagents. RPMI 1640 (Cat. No. 11875-093), DMEM (Cat. No. 10313-021), 0.25% trypsin (Cat. No. 25200-056), PBS (Cat. No. 10010-023), HEPES (Cat. No. 15630-080) and glutamine (Cat. No. 25030-081) were procured from Gibco Laboratory (Gaithersburg, MD). DMEM low glucose (Cat. No. D6046-500ML), high glucose (Cat. No. D6429-500ML) media, MitoTEMPO (Cat. No. SML0737), antimycin A (Cat. No. A8674-25MG), Triton X-100 (Cat. No. T-8787), bovine serum albumin (Cat. No. A9647-100G), Tween 20 (Cat. No. P2287), d-mannitol (Cat. No. M4125-500G), dimethyl sulfoxide (Cat. No. D2650-100ml) and Cell Proliferation ELISA, BrdU (Cat. No. 11 647 229 001) were purchased from Millipore Sigma (Burlington, MA). Fetal bovine serum (Cat. No. SH30071.03HI) was obtained from HyClone Laboratories (Logan, UT). CellTiter-Blue® cell viability assay (Cat. No. G8081) was acquired from Promega (Madison, WI). Corning® Matrigel® Basement Membrane Matrix (Cat. No. 354234) was purchased from Corning Inc. (Corning, NY). The AlphaTrak blood glucose monitoring system (Cat. No. 32107K) was obtained from ADW Diabetes (Pompano Beach, FL). Cell migration plates (CIM plate-16, Cat. No. 5665817001) were purchased from Agilent Technologies (Santa Clara, CA). The RNeasy Plus Mini (Cat. No. 74134) and DNeasy Blood & Tissue kits (Cat. No. 69506) are products from QIAGEN (Germantown, MD). Olaparib (Cat. No. S1060), berzosertib (Cat. No. S7102), AZD7762 (Cat. No. S1532), etoposide (Cat. No. S1225), and sonidegib (Cat. No. S2151) are from Selleckchem (Houston, TX). MitoSOX™ Red Mitochondrial Superoxide Indicator (Cat. No. M36008) and 0.5M EDTA (Cat. No. AM9260G) are products of Invitrogen (Waltham, MA). Anti-gamma H2A.X (phospho S139) (Cat. No. ab11174) and goat anti-rabbit IgG H&L (Alexa Fluor® 488) (Cat. No. ab150077) antibodies are from Abcam (Cambridge, MA) and the anti-53BP1 antibody (Cat. No. NB100-304) was

obtained from Novus Biologicals (Littleton, CO). ProLong™ Gold Antifade Mountant with DAPI (Cat. No. P36941), 8-well glass chamber slides (Cat. No. 154941), Lipofectamine™ LTX Reagent with PLUS™ Reagent (Cat. No. 15338100), 4% paraformaldehyde (Cat. No. AAJ61899AK) and TRIzol Reagent (Cat. No. 15596018) were procured from Thermo Fisher Scientific (Waltham, MA). N-(1-carboxymethyl)-L-lysine was obtained from Cayman Chemical, Ann Arbor, MI. pLCN DSB Repair Reporter (Cat. No. 98895) and pCBASceI (Cat. No. 26477) were procured from Addgene (Watertown, MA). Custom lentivirus production for plasmid pLCN DSB Repair Reporter and DNA preparation for plasmid pCBASceI were performed by GeneCopoeia (Rockville, MD). TRULI, a Lats1/2 kinase inhibitor, was a gift from Lalage Wakefield (NCI, Bethesda, MD).

Collection of human breast tumors and patient data. Samples of fresh-frozen tumor were prepared by a pathologist immediately after surgery, following established protocols, and stored at -80° C. Demographic, clinical and pathological information (e.g., hormone receptor status) was obtained from medical records and pathology reports. Information on diabetes and the use of medication was also obtained from the medical records. Disease staging was performed according to the tumor–node–metastasis (TNM) system of the American Joint Committee on Cancer/ the Union Internationale Contre le Cancer (AJCC/UICC). The Nottingham system was applied to determine tumor grade. Collection of both biospecimens and the clinical and pathologic information was approved by the University of Maryland Institutional Review Board (UMD protocol no. 0298229). The research was also reviewed and approved by the NIH Office of Human Subjects Research Protections (OHSRP no. 2248). Informed written consent was obtained from all patients.

Patient samples. As outlined in Supplemental Figure 1C, we initially worked with a cohort of 100 female patients (40 individuals with diabetes and 60 without diabetes), from whom we generated our RNA sequencing and metabolome data. In the later part of this study, to perform WES and IHC staining, we added additional patients. For WES, the number of patients in the analysis is 116 (35 female and 3 male patients with diabetes + 7 female patients who developed diabetes after their breast cancer surgery + 67 female and 4 male patients without a diabetes diagnosis; 35 female patients with diabetes and 60 female patients without diabetes overlapped with the initial cohort). For IHC, the number of patients is 105. Among the 105, 29 were patients with diabetes, which is a subset of the 40 diabetes patients in the initial dataset, and 76 patients did not have a diagnosis of diabetes (included all patients from the initial cohort).

Orthotopic tumor growth in mice with diabetes/hyperglycemia. Six mice were housed per cage and given autoclaved food and water ad libitum. All of them received the NIH-31 Open Formula diet. We orthotopically injected ER-negative breast cancer cells (MDA-MB-231, MDA-MB-468 and Hs578T) into the mammary fat pad of mice for tumor growth (n = 6 per experimental group). We did not use ER-positive cell lines because of the difficulty to obtain tumor xenografts with the slow-growing ER-positive cell lines. To establish xenografts, 8 weeks old diabetic and non-diabetic mice were injected unilaterally with 5×10^6 cells in 100 µL of Matrigel/PBS (50:50) into the fourth abdominal fat pad by subcutaneous injection. Tumor growth was monitored weekly and measured externally using Vernier Calipers. The tumor-growing mice were sacrificed at 5 weeks (for MDA-MB-231 xenografts), 6 weeks (for MDA-MB-468 xenografts), or 8 weeks (for Hs578T xenografts) after the initial inoculation of the breast

cancer cells into the fat pads. Tumors were obtained and the weight was recorded. Tumors were immediately frozen in liquid nitrogen for subsequent RNA and DNA extraction and the analysis of the tumor metabolome. In each of the control groups, only 5 out of 6 mice developed tumor xenografts (83%) whereas tumor xenograft growth occurred in all Akita mice. Among tumor-bearing Akita mice, 5 animals had to be sacrificed prior to the end point because of deteriorating health (n = 2, MDA-MB-231 and Hs578T; n = 1, MDA-MB-468). The protocol for this experiment was reviewed and approved by the NCI Animal Care and Use Committee (Animal Study Protocol Number LHC-013) and experiments were performed in accordance with principles outlined in the Guide for the Care and Use of Laboratory Animals at NIH. At the end of the experiments, but before sacrificing the animals, blood was collected, and glucose levels were measured using the AlphaTrak blood glucose monitoring system. Animals were also assessed for presence of metastatic lesions in the lung when the tumor xenografts were surgically removed for molecular analysis.

Histological evaluation of metastasis. Two pathologists (Yfantis, Flis) reviewed the histology of the metastatic lesions derived from the MDA-MB-231 xenografts. Both confirmed the presence of metastatic carcinoma in the gastrointestinal tract and spleen of xenograft-bearing mice.

BrdU incorporation assay assessing inhibition of cell proliferation by DNA repair pathway inhibitors. Cell proliferation was measured with the BrdU cell proliferation kit from Sigma-Aldrich (Cat. No. 11647229001) following vendor’s instructions. In brief, cells were plated in 96 well plates (5,000/well). The next day, cells were exposed to various concentrations (1 nM to 50 µM) of DNA repair pathway inhibitor drugs (AZD7762, berzosertib, etoposide, and olaparib) in 100 µl of low (5 mM) or high glucose (25 mM) medium in a humidified atmosphere at 37°C.

After 24 hours, 10 µl of BrdU labeling solution (100 µM BrdU stock) was added to each well (final concentration: 10 µM BrdU) and cells were incubated for an additional 24 hours at 37°C. Next, labeling medium was removed from adherent cells by tapping off or using suction. Cells were fixed with 200 µl/well FixDenat (provided in the kit) and incubated for 30 minutes at 15 to 25°C. The FixDenat solution was decanted, and cells were incubated for 2 hours with 100 µl/well Anti-BrdU-POD working solution. Next, the antibody conjugate was removed, and wells were rinsed three times with 200 to 300 µl/well washing solution (1x PBS). Finally, washing solution was removed and 100 µl/well substrate solution was added. The absorbance of the samples was measured in an ELISA reader at 370 nm (reference wavelength approximately 492 nm).

Cell viability assay. In brief, cells were plated in 96 well plate (2,000/well) in culture medium. The next day, media was removed, and cells were cultured in 200 µl of either low or high glucose media. After additional 48 hrs. assay plates were removed from 37°C incubator and 20 µl of CellTiter-Blue® Reagent was added to each well. Next, plates were incubated using standard cell culture conditions for 1–4 hours. Finally, the fluorescence reading was recorded at 560/590 nm, and cell viability was compared between cells grown in low and high glucose. For other experiments, the assay was applied when breast cancer cells were additionally treated with either DNA repair pathway inhibitors (AZD7762, berzosertib, etoposide, and olaparib) or a hedgehog signaling inhibitor (sonidegib).

Migration and invasion assay. Briefly, for migration assay, cells were plated onto uncoated CIM-Plate 16. 100 µl of serum-free media containing 50,000 cells under low or high glucose conditions was added to the upper chamber for cell plating and the lower chamber was filled

with 10% FBS cell culture media. For the invasion assay, the membrane in the top chamber of the CIM plate was coated with 30 µl of a 1:20 dilution of Matrigel (BD Biosciences, San Jose, CA). Loaded CIM-Plates were placed into the xCelligence analyzer and electrical impedance was measured every 15 min over a 24-hour period. The migration and invasion indices were determined as per manufacturer's instructions. For some migration experiments, a ROS scavenger, Mitotempo, was added to the cell culture medium.

Quantification of the mesenchymal phenotype in cell culture. To examine the development of a mesenchymal morphology, $3x10^6$ cells (MDA-MB-231 and Hs578T) were plated in T25 flask and cultured in low or high glucose medium for 48 hours. Images of cells were taken using a phase contrast microscope (Olympus IX51) at ×200 magnification. These images were processed using the software ImageJ (version 1.50i at http://imagej.nih.gov/ij/). Cells treated with high glucose developed more frequently an elongated, mesenchymal phenotype when compared to low glucose cultured cells. For quantification purpose, the cell length was measured using ImageJ software. Experiments were performed in triplicate and average cell length in each group was calculated (per 100 evaluated cells) using cells from representative fields across experiments.

Stemness reporter assay. To evaluate induction of a cancer stem cell phenotype under hyperglycemic condition, we applied a stem cell reporter assay using modified MDA-MB-231 cells, as described previously (1). Briefly, MDA-MB-231-LM2 cells constitutively express a reporter construct in which six concatenated repeats of a composite SOX2/OCT4 response element (SORE6) from the proximal human NANOG promoter are coupled to a minimal cytomegalovirus (CMV) promoter, to drive expression of a fluorescent reporter gene for stem

cell signaling. In the experiment, on day 1, MDA-MB-231-LM2 cells (both vector control and SORE6 reporter construct harboring cells) were plated on a 24 well plate (25000 cells/well). On day 2, the medium was decanted and replaced with fresh low (5 mM) or high (25 mM) glucose DMEM media supplemented with minimal serum (0.1%). In some of the experiments, 5 µM of a positive control compound (TRULI, a Lats1/2 kinases inhibitor) was added to the MDA-MB-231-LM2_SORE6 cells when cultured +/- high glucose. For imaging, the plates were kept inside an IncuCyteFLR live-cell imaging system (Essen Instruments) for 48 hrs. IncuCyteFLR is equipped with a 20X objective lens, which can take high-definition phase-contrast and green fluorescence images in real time. The obtained images were then analyzed using the IncuCyte software. The number of mCherry and GFP positive cells were determined using the same software.

Measurement of mitochondrial ROS. Briefly, cells (MDA-MB-231 and Hs578T) were seeded in a T25 flask at 4×10^5 cells per well in regular culture media. The next day, culture media were switched to either low or high glucose media and incubated for 48 hours to determine if high glucose induces mitochondrial ROS. To measure mitochondrial ROS production, MitoSOX Red mitochondrial superoxide indicator (Molecular Probes, M36008) was applied to live cells, following standard protocols. Cultured cells were washed with PBS and treated with 5 µM MitoSOX Red for 30 minutes at 37°C without light exposure. As a positive control, cells were treated first with 10 µM antimycin for 15 min before MitoSOX Red was added. Cells were then trypsinized and washed with PBS, and finally resuspended in PBS for the analysis by flow cytometry. Samples were analyzed in the flow cytometer (SA3800 Spectral Analyzer, Sony Corporation, Tokyo, Japan) to measure oxidized MitoSOX Red. Data analysis was performed using FlowJo version 10.7.1.

Immunofluorescence microscopy to quantify DNA damage. MDA-MB-231 and Hs578T cells were plated (10,000 cells/well) in an 8 well glass chamber slides (Thermo Fisher Scientific, Waltham, MA). Starting the next day, cells were cultured in either low or high glucose media for 48 hours to determine if high glucose increases DNA damage. To assess damage, cells were fixed after 48 hours of culture with 4% paraformaldehyde for 20 min, subsequently washed with PBS. Next, permeabilization was done by incubating the cells with 0.25% Triton X-100 (Cat. No. T-8787, Millipore Sigma, Burlington, MA) in PBS for 10min. Then cells were washed 3-times with PBS. A blocking step to reduce unspecific antibody binding was performed by incubating cells with blocking reagent [1%BSA in PBS and 0.1% Tween 20 (Millipore Sigma, Burlington, MA] for 30 min. Then cells were incubated overnight with primary antibodies (either anti-gamma H2A.X or 53BP1 antibodies) at a dilution of 1:1000 in blocking reagent. The next day, the solution was decanted, and cells were washed 3-times with PBS. Next, cells were incubated with anti-rabbit secondary antibody [goat anti-rabbit IgG H&L (Alexa Fluor® 488)] in blocking reagent at a dilution of 1:400 for 1hr in room temperature. Cells were washed 3-times in PBS followed by mounting them with ProLong™ Gold Antifade Mountant containing DAPI (Thermo Fisher Scientific, Waltham, MA). Slides were then dried in the dark overnight and stored at 4° C until imaging by confocal microscopy. Images were taken on a Nikon confocal Ti2 with Yokagawa CSU-X1 spinning disk and a photometrics Prime 95B camera using a Nikon water 60x 1.2-NA Plan Apo objective. Images were captured using the Elements version 5.20.00 software. Images were processed with ImageJ (release 1.50i; http://imagej.nih.gov/ij).

Measurement of DNA repair capacity in breast cancer cells under hyperglycemic condition.

This assay uses a fluorescence-based reporter system (pLCN DSB Repair Reporter) that allows the quantitative measurement of NHEJ in cells through the repair of two inverted ISce1 cuts in pCBASceI plasmid. Repair through NHEJ leads to expression of GFP, which can be monitored by FACS analysis. In brief, MDA-MB-231 and Hs578T cells were plated in a 6 well plate ($2x10^5$ cells/well). The next day, cells were transduced with lentiviral particles containing the pLCN DSB Repair Reporter. After 48 hours, the cell culture medium was decanted, and cells were transfected with pCBASceI plasmid (2.5 µg/well) using Lipofectamine® LTX DNA Transfection Reagents according to the manufacturer's instruction. Cells without pCBASceI served as control. After 24 hours, cells were cultured in either low or high glucose cell culture medium for another 48 hours. Then, cells then were trypsinized and resuspended in 25 mM HEPES pH 7, 1% (v/v) FBS, 1x PBS, 2 mM EDTA and subjected to flow cytometry analysis with a ID7000™ Spectral Cell Analyzer (Sony Corporation, Tokyo, Japan).

Metabolome analysis of human tumors and xenografts. The metabolome of fresh-frozen human tumors was investigated using untargeted metabolic profiling performed by the service provider, Metabolon, Inc. (Morrisville, NC). We previously described the methodology (2). The mass-spectroscopy-based and fully automated platform measures a total of up to 830 metabolites. We analyzed the metabolome of breast tumors from women with (n=40) and without (n=48) diabetes. Additionally, metabolomic profiling was performed in xenograft tumors grown from MDA-MB-231, MDA-MB-468, and Hs578T cells in diabetic and non-diabetic mice, and in serum samples of Akita mice bearing MDA-MB-468 tumor xenografts. For the metabolome analysis, frozen human or xenograft tumors and mouse serum samples were shipped to Metabolon for *in-house* extraction and measurements of the metabolites.

Metabolon provided both raw and processed measurement data. The metabolomics data have been deposited in the Open Science Framework (https://osf.io) at https://osf.io/h73rf/?view_only=.

Transcriptome analysis of human tumors, xenografts, and cultured cells. Following RNA isolation from human tumors, the integrity of isolated RNA was evaluated with the Agilent 2100 Bioanalyzer (Agilent Technologies). RNA sequencing (RNA-Seq) was performed at the Sequencing Facility, Leidos Biomedical Research, Inc., Frederick National Laboratory for Cancer Research, using standard protocols. Briefly, 500 ng of total RNA was used for library preparation with TruSeq stranded mRNA Prep (Illumina). Sequencing was performed on an Illumina NovaSeq system. For each sample, we generated approximately 50 million paired-end reads at 101-bp length. Reads were trimmed for both adapters and low-quality bases using Trimmomatic software and then aligned with the reference human hg19 genome and gene annotation from the Ensembl database (v70) using Tophat software. RNA mapping statistics were calculated using the Picard software, and the average uniquely aligned reads were approximately 90% for each sample. Differential expression analysis was done in R Studio version 3.5 (R Foundation for Statistical Computing; http://www.r-project.org/) using DESeq2_1.24.0 package (3). Additionally, we used the R/Bioconductor package edgeR to generate CPM_TMM normalized counts. Briefly, starting with the data in the raw count file, any gene that did not have a CPM value of 0.5 in two or more samples was filtered out. calcNormFactors was then used to calculate the normalization factors for the remaining genes using the TMM normalization method. CPM was then calculated while using the TMM normalization factors. This CPM_TMM normalized file was then imported to Qlucore Omics Explorer 3.7 to generate PCA plots, fold change, volcano plots, and heatmaps(4,

5). RNA-Seq data for the breast tumors were deposited in the NCBI's Gene Expression Omnibus (GEO) database under accession number (GSE202922). Sequencing of RNA from the human xenografts (MDA-MB-231, MDA-MB-468 and HS578T +/- diabetes) was performed as described above following isolation of RNA with the RNeasy Plus Mini Kit. Sequencing was performed on an Illumina NextSeq sequencer. Data normalization and subsequent analysis was performed as mentioned above. The RNA-Seq data for xenografts were deposited in the NCBI's Gene Expression Omnibus (GEO) database under accession number (GSE202599). Additional RNA sequencing was performed for ER-positive (MDA-MB-175, ZR7530, HCC1500) and ER-negative (MDA-MB-157, MDA-MB-231, MDA-MB-468) breast cancer cells. Cells were cultured under low (5 mM) and high (25 mM) glucose. For each experimental group there were four replicates. In brief, 5×10^5 cells in a T25 flask were treated with low or high glucose for 48 hours before RNA was isolated. RNA isolation was done using RNeasy Plus Mini Kits from QIAGEN. Integrity of isolated RNA was evaluated with the Agilent 2100 Bioanalyzer (Agilent Technologies), and sequencing was performed on an Illumina HiSeq machine. RNA-Seq data for the cell lines were deposited in the NCBI's Gene Expression Omnibus (GEO) database under accession number (GSE202595, GSE202598, and GSE236420).

Metabolomic and transcriptomic data integration. Metabolomic and transcriptomic data were integrated using a correlation-based integration approach (6) followed by GSEA (7). The goal of this analysis was to identify the transcriptomic programs underlying the metabolomic contrasts in xenografts due to diabetes. For each expressed gene, we determined a score defined as the median signed correlation across all differentially abundant metabolites, i.e. $\text{median}[\text{cor}(\text{gene},\text{metab})*\text{sign}(\log(FC_{metab}))]$, where cor(gene,metab) is the Spearman's

correlation between the expression of the target gene and that of a differentially abundant metabolite across the 24 xenograft samples (MDA-MB-231, MDA-MB-468, Hs578T), and where FC_{metab} is the combined metabolite fold difference measured between diabetic and non-diabetic mice across the three strains. We restricted this analysis to the 67 metabolites that were consistently increased (n = 53) or decreased (n = 14) in the tumors of the diabetic Akita mice; see **Table S2**. All measured genes where thus rank-ordered by score; on the resulting ranked gene list, GSEA was performed using the R package "fgsea" (v. 1.20.0) (8) and reference gene collection "Hallmarks" from the Molecular Signatures Database MSigDB (v. 7.4) (9). A customized implementation in R was developed in-house to add robustness to the GSEA analysis; since GSEA's enrichment estimates (and statistical significance) are stochastic, our software embeds GSEA in a Monte Carlo algorithm that performs 100 iterations and chooses significant pathways based on the total statistical ensemble. We report only pathways that appeared significant (based on adjusted $P < 0.05$) in more than 80% of the iterations (see **Table S8**).

Whole exome sequencing (WES). DNA was extracted from breast tumors of patients with (n=38) and without (n=71) diabetes, or from patients who developed diabetes on follow-up (n=7). Extractions were done with the QIAGEN DNeasy blood & tissue kit. DNA quality was checked by the NIH Genomics Core using the Agilent Genomic DNA Screen tape assay. The extracted DNA samples were used for library preparation using SureSelect V6+ UTR-postcapture with 89Mb of targeted exonic sequence. The sequencing library was prepared by random fragmentation of the DNA or cDNA sample, followed by 5' and 3' adapter ligation. For some samples, "tagmentation" that combines the fragmentation and ligation reaction into a

single step was used because it greatly increases the efficiency of the library preparation process. Adapter-ligated fragments were PCR amplified and gel purified. For cluster generation, the library was loaded into a flow cell where fragments were captured on a lawn of surface-bound oligos complementary to the library adapters. Each fragment was then amplified into distinct, clonal clusters through bridge amplification. After completing the cluster generation, templates were ready for sequencing. Illumina SBS technology was utilized as a proprietary reversible terminator-based method that detects single bases as they are incorporated into DNA template strands. As all 4 reversible, terminator-bound dNTPs are present during each sequencing cycle, natural competition minimizes incorporation bias and greatly reduces raw error rates compared to other technologies. The result is highly accurate base-by-base sequencing that virtually eliminates sequence-context-specific errors, even within repetitive sequence regions and homopolymers. An Illumina sequencer was used to generate raw images utilizing sequencing control software for system control and base calling through an integrated primary analysis software called RTA (Real Time Analysis). The BCL (base calls) binary was converted into FASTQ utilizing Illumina package bcl2fastq. The WES raw data for the tumors have been deposited in the SRA database under accession number (PRJNA840859).

Variant calling was performed using Mutect2 in tumor-normal mode (10) following the best practices guidelines for exome-seq analysis provided by the GATK authors (11). Variants were hard-filtered for quality, annotated with functional and consequence prediction using Ensemble's Variant Effect Predictor (12) (VEP v. 92) and converted to Mutation Annotation Format (MAF) using the vcf2maf tool (v. 1.6.16). MAF files for individual samples were concatenated into a combined MAF file spanning the full cohort for downstream analysis. Trinucleotide frequency

patterns were extracted with maftools (13) (v. 1.8.10). Reference mutational signatures were obtained from the Catalogue Of Somatic Mutations In Cancer (COSMIC), version v3.1 (June 2020; COSMIC release v91) (14), as well as from the Compendium of Mutational Signatures of Environmental Agents (May 2019) (15). This information was fed into deconstructSigs (16) (v. 1.8.0) to generate subject/signature weights from the non-negative least squares mapping of individual samples against the reference signatures. These weights are determined such that the reconstructed tumor sample matrix minimizes a given error threshold. To reduce false positives, some corrections can be applied to the fitting approach; for example, deconstructSigs uses forward selection to estimate a minimal number of signatures and removes a signature's contribution to a sample if it accounts for less than 6% of the sample's mutations. Limitations of deconstructSigs and other mutational signature methods have been discussed and benchmarked elsewhere (17). For each mutational signature compendium, the subject/signature weight matrix was obtained; then, signatures with distinct prevalence between diabetic and non-diabetic groups were identified by the criterion of $P < 0.05$ in the Wilcoxon test performed between their weight distributions. This comparative analysis was carried out for all diabetics and non-diabetics, as well as for ER-negative and ER-positive sub-cohorts. All findings including FDR are reported in **Table S10**. Additionally, an oncoplot was generated to visualize the top mutated genes across subjects ordered by diabetes group. After keeping only nonsilent mutations, genes were selected as those mutated > 5% of the samples. The oncoplot was generated using the R package "ComplexHeatmap" (v. 2.12.1) (18).

Gene Set Enrichment Analysis (GSEA) and further validation using pathway "activity" scores.

RNA sequencing-based transcriptome data from human breast tumors, tumor xenografts and cell lines were used for GSEA and pathway analysis. GSEA was performed as previously described (9) using the Qlucore Omics Explorer 3.7 software. Briefly, CPM_TMM normalized RNA-seq counts were imported to Qlucore. For the transcripts with multiple expression values, the average expression value of all transcripts was considered for the analysis. The fold change of all genes between two groups was calculated using the two-group comparison (t- test). The entire gene list together with fold change was imported into the GSEA analysis using the default Qlucore GSEA settings (Min Match Set Size: 15, Max Match Set Size: 500, Permutation: 1000, Permutation Methods: samples, Enrichment weight: 1). Hallmark and KEGG gene sets (n = 50 and 186, respectively) were selected within MSigDB as references for pathway analysis. Gene sets or pathways with an FDR < 0.25 were included in our data presentation following Broad Institute guidelines. According to the GSEA website (https://software.broadinstitute.org/cancer/software/gsea/wiki/index.php/FAQ), an FDR of 25% is reasonable in the setting of discovery.

To supplement and validate the GSEA gene set/pathway discovery approach, we generated additional pathway scores based on z-scores. For each gene in each target pathway, expression was transformed by log2(x+1) followed by z-score transformation across all samples. Two approaches to summarize pathway-level expression are (i) taking the first principal component from Principal Component Analysis (“PC1”) and (ii) taking the average expression across all genes in the target pathway (“AVG”). Since principal components are sign-indeterminate, pathway scores were defined as PC1*sign(cor(PC1,AVG)), where cor() denotes

Pearson's correlation estimate. The statistical significance of pathway score differences between diabetic and non-diabetic cohorts was assessed via Wilcoxon tests.

Single Sample Gene Set Enrichment Analysis (ssGSEA). Pathway-level expression scores were obtained using the ssGSEA method, which computes an enrichment score for each gene set and individual sample by using the difference in empirical cumulative distribution functions of gene expression ranks inside and outside the target gene set (19). ssGSEA was implemented via the R package "GSVA" (v. 1.44.5) (20). To perform pathway-level differential expression analysis comparing diabetic with non-diabetic samples, we fitted a multivariable linear regression model to each target gene set using the formula "score ~ Db + age + BMI + race + stage + ER", which allows to determine the statistical significance of diabetes status (Db) affecting differential expression while controlling for multiple demographic and clinical covariates. Linear regression was implemented via the base R package "stats" (v. 4.2.1). After removing the ER status as covariate, similar analyses were carried out separately within the ER+ and ER- sub-cohorts. To visually compare adjusted pathway-level expression, we removed Db as covariate and plotted the residuals from the resulting multivariable regression fits.

Transcriptome analysis of N-(1-carboxymethyl)-L-lysine (CML) exposed breast cancer cells. Briefly, 5×10^5 MDA-MB-231 cells were cultured in a T25 flask and treated with 1 µM CML for 48 hours in low glucose (5mM) medium (n = 4). PBS was added to the control cells. After 48 hours, RNA was isolated using RNeasy Plus Mini Kits from QIAGEN. Integrity of isolated RNA was evaluated with the Agilent 2100 Bioanalyzer (Agilent Technologies), and sequencing was performed on an Illumina HiSeq machine. The RNA-Seq data are deposited in the NCBI's Gene Expression Omnibus (GEO) database under accession number (GSE202597).

Gene expression and metabolome analysis with covariate adjustments. Diabetic and non-diabetic breast cancer patients showed differences in demographic and clinical characteristics. Hence, we adjusted for key differences when generating gene lists comparing tumors from diabetic and non-diabetic patients. RNA-Seq differential expression analysis comparing diabetic with non-diabetic patient samples was performed by fitting a multivariable linear regression model to each metabolite or gene using the formula "expression ~ Db + age + BMI + race + stage + ER", which allows to determine the statistical significance of Db affecting differential expression while controlling for multiple demographic and clinical covariates. Linear regression was implemented via the base R package "stats" (v. 4.2.1). By removing ER status as covariate, similar analyses were carried out separately within the ER+ and ER- sub-cohorts. Following the same steps, we generated a second list for differential metabolite but without further stratification by tumor ER status. The multivariate adjusted data for RNAseq and metabolome analysis are provided in 2 separate resource excel files (Supplementary resource file_ Multivariate RNAseq and Supplementary resource file_Multivariate_Metabolome).

MetaboAnalyst. We performed a pathway enrichment analysis with metabolites as input applying MetaboAnalyst (V5.0), which is a platform dedicated to metabolomics data analysis via a user-friendly and web-based interface (21). In brief, we imported the metabolite list to the MetaboAnalyst (V5.0) platform and performed the over representation analysis using the Small Molecule Pathway Database (SMPDB). Enrichment Ratio is computed by observed Hits/ Expected hits.

References

1. Tang B, Raviv A, Esposito D, Flanders KC, Daniel C, Nghiem BT, et al. A flexible reporter system for direct observation and isolation of cancer stem cells. *Stem Cell Reports.* 2015;4(1):155-69.
2. Terunuma A, Putluri N, Mishra P, Mathe EA, Dorsey TH, Yi M, et al. MYC-driven accumulation of 2-hydroxyglutarate is associated with breast cancer prognosis. *JClin Invest.* 2014;124(1):398-412.
3. Tang W, Putluri V, Ambati CR, Dorsey TH, Putluri N, and Ambs S. Liver- and Microbiome-derived Bile Acids Accumulate in Human Breast Tumors and Inhibit Growth and Improve Patient Survival. *Clin Cancer Res.* 2019;25(19):5972-83.
4. Lee AJ, Fraser E, Flowers B, Kim J, Wong K, Cataisson C, et al. RAS induced senescence of skin keratinocytes is mediated through Rho-associated protein kinase (ROCK). *Mol Carcinog.* 2021;60(12):799-812.
5. Rossmann L, Bagola K, Stephen T, Gerards AL, Walber B, Ullrich A, et al. Distinct single-component adjuvants steer human DC-mediated T-cell polarization via Toll-like receptor signaling toward a potent antiviral immune response. *Proc Natl Acad Sci U S A.* 2021;118(39).
6. Cavill R, Jennen D, Kleinjans J, and Briede JJ. Transcriptomic and metabolomic data integration. *Brief Bioinform.* 2016;17(5):891-901.
7. Mootha VK, Lindgren CM, Eriksson KF, Subramanian A, Sihag S, Lehar J, et al. PGC-1alpha-responsive genes involved in oxidative phosphorylation are coordinately downregulated in human diabetes. *Nat Genet.* 2003;34(3):267-73.
8. Korotkevich G, Sukhov V, Budin N, Shpak B, Artyomov MN, and Sergushichev A. Fast gene set enrichment analysis. *bioRxiv.* 2021:060012.
9. Subramanian A, Tamayo P, Mootha VK, Mukherjee S, Ebert BL, Gillette MA, et al. Gene set enrichment analysis: a knowledge-based approach for interpreting genome-wide expression profiles. *Proc Natl Acad Sci USA.* 2005;102(43):15545-50.
10. Cibulskis K, Lawrence MS, Carter SL, Sivachenko A, Jaffe D, Sougnez C, et al. Sensitive detection of somatic point mutations in impure and heterogeneous cancer samples. *Nat Biotechnol.* 2013;31(3):213-9.
11. Van der Auwera GA, Carneiro MO, Hartl C, Poplin R, Del Angel G, Levy-Moonshine A, et al. From FastQ data to high confidence variant calls: the Genome Analysis Toolkit best practices pipeline. *Curr Protoc Bioinformatics.* 2013;43:11 0 1- 0 33.
12. McLaren W, Gil L, Hunt SE, Riat HS, Ritchie GR, Thormann A, et al. The Ensembl Variant Effect Predictor. *Genome Biol.* 2016;17(1):122.
13. Mayakonda A, Lin DC, Assenov Y, Plass C, and Koeffler HP. Maftools: efficient and comprehensive analysis of somatic variants in cancer. *Genome Res.* 2018;28(11):1747-56.
14. Tate JG, Bamford S, Jubb HC, Sondka Z, Beare DM, Bindal N, et al. COSMIC: the Catalogue Of Somatic Mutations In Cancer. *Nucleic Acids Res.* 2019;47(D1):D941-D7.
15. Kucab JE, Zou X, Morganella S, Joel M, Nanda AS, Nagy E, et al. A Compendium of Mutational Signatures of Environmental Agents. *Cell.* 2019;177(4):821-36 e16.
16. Rosenthal R, McGranahan N, Herrero J, Taylor BS, and Swanton C. DeconstructSigs: delineating mutational processes in single tumors distinguishes DNA repair deficiencies and patterns of carcinoma evolution. *Genome Biol.* 2016;17:31.
17. Maura F, Degasperi A, Nadeu F, Leongamornlert D, Davies H, Moore L, et al. A practical guide for mutational signature analysis in hematological malignancies. *Nat Commun.* 2019;10(1):2969.
18. Gu Z, Eils R, and Schlesner M. Complex heatmaps reveal patterns and correlations in multidimensional genomic data. *Bioinformatics.* 2016;32(18):2847-9.
19. Barbie DA, Tamayo P, Boehm JS, Kim SY, Moody SE, Dunn IF, et al. Systematic RNA interference reveals that oncogenic KRAS-driven cancers require TBK1. *Nature.* 2009;462(7269):108-12.

20. Hanzelmann S, Castelo R, and Guinney J. GSVA: gene set variation analysis for microarray and RNA-seq data. *BMC Bioinformatics.* 2013;14:7.
21. Xia J, Psychogios N, Young N, and Wishart DS. MetaboAnalyst: a web server for metabolomic data analysis and interpretation. *Nucleic Acids Res.* 2009;37(Web Server issue):W652-60.
